# Pushing the Electrochemical Performance Limits of Polypyrrole Toward Stable Microelectronic Devices

**DOI:** 10.1007/s40820-023-01027-3

**Published:** 2023-02-13

**Authors:** Muhammad Tahir, Liang He, Lihong Li, Yawei Cao, Xiaoxia Yu, Zehua Lu, Xiaoqiao Liao, Zeyu Ma, Yanlin Song

**Affiliations:** 1 Key Laboratory of Green Printing, CAS Research/Education Centre for Excellence in Molecular Sciences, Institute of Chemistry Chinese Academy of Sciences (ICCAS), Beijing, 100190 People’s Republic of China; 2 School of Mechanical Engineering, Sichuan University, Chengdu, 610065 People’s Republic of China; 3 Med+X Center for Manufacturing, West China Hospital, Sichuan University, Chengdu, 610041 People’s Republic of China; 4 School of Chemistry and Biological Engineering, University of Science and Technology Beijing, Beijing, 100083 People’s Republic of China

**Keywords:** Microsupercapacitor, Skin-compatible device, Strain sensor, Flexible electronics

## Abstract

Conducting polymers have achieved remarkable attentions owing to their exclusive characteristics, for instance, electrical conductivity, high ionic conductivity, visual transparency, and mechanical tractability. Surface and nanostructure engineering of conjugated conducting polymers offers an exceptional pathway to facilitate their implementation in a variety of scientific claims, comprising energy storage and production devices, flexible and wearable optoelectronic devices. A two-step tactic to assemble high-performance polypyrrole (PPy)-based microsupercapacitor (MSC) is utilized by transforming the current collectors to suppress structural pulverization and increase the adhesion of PPy, and then electrochemical co-deposition of PPy-CNT nanostructures on rGO@Au current collectors is performed. The resulting fine patterned MSC conveyed a high areal capacitance of 65.9 mF cm^−2^ (at a current density of 0.1 mA cm^−2^), an exceptional cycling performance of retaining 79% capacitance after 10,000 charge/discharge cycles at 5 mA cm^−2^. Benefiting from the intermediate graphene, current collector free PPy-CNT@rGO flexible MSC is produced by a facile transfer method on a flexible substrate, which delivered an areal capacitance of 70.25 mF cm^−2^ at 0.1 mA cm^−2^ and retained 46% of the initial capacitance at a current density of 1.0 mA cm^−2^. The flexible MSC is utilized as a skin compatible capacitive micro-strain sensor with excellent electromechanochemical characteristics.

**Supplementary Information:**

The online version contains supplementary material available at 10.1007/s40820-023-01027-3.

## Highlights

- Optimizing the current collectors with rGO and the addition of CNT in PPy, and the structural pulverization is prevented significantly.
- Areal capacitances of 65.9 to 70 mF cm^−2^ are achieved for PPy-CNT@rGO microsupercapacitors (MSCs) with 79% capacitance retention after 10,000 cycles.
- The MSC-derived capacitive micro-strain sensor is capable of biosignals detection.
- The micro strain sensor is capable of detecting wide levels of applied strain with short response/recovery time and excellent reliability.

**Supplementary Information:**

The online version contains supplementary material available at 10.1007/s40820-023-01027-3.

## Graphical abstract

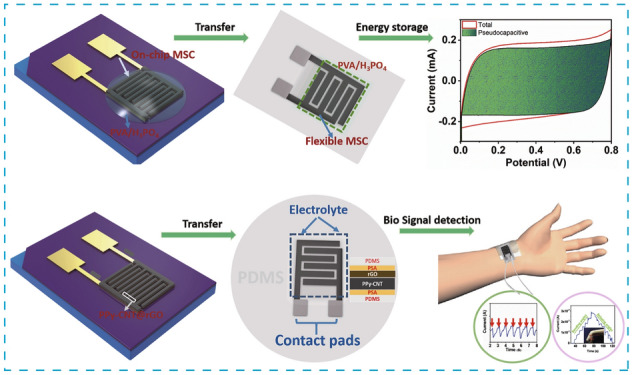

## Introduction

The fast-increasing need for portable microelectronic devices, such as microsensors, micro/nanorobots, micro-biomedical equipment (e.g., enological stimulators), and radio-frequency identifiers, has demanded the construction and design of high-performance micro-energy storage devices with superficial charge storage characteristic and high compatibility with miniaturized electronic devices [1]. Within this perspective on-chip characteristic, microsupercapacitors (MSCs) have provoked excessive attentions due to their awesome performance with their long-term cycling stability, rapid charge/discharge, high power density, high-frequency response, and attractive reversibility [2]. Currently, significant research efforts are devoted to enhancing the energy and power densities of MSCs by assembling a state-of-the-art nanostructured architecture of materials with superior surface area and enlightened architectures [3]. Therefore, out of advanced materials consisting of nanostructures, carbonaceous materials and their derivatives such as graphene [4], onion structure carbon [5], carbon nanotubes (CNTs) [6], and carbide derivative carbon [7] have gained much attention due to their excellent double-layer charge storage behavior, long-term cycling stability, and suitable operating voltage window. Despite the important progress in nanostructured carbon materials, the construction of high-energy MSCs remains a great challenge for researchers.

Electrochemically polymerized conducting polymers (EPCPs), rising promising components in pseudocapacitive materials, show great potential for constructing supercapacitors [8–11], rechargeable batteries [12–14], optoelectronic devices [15–17], transistors [18–20], stretchable circuits [21], and light emitting diodes [22–24], due to their high electrical conductivity, relatively satisfactory specific capacitance, transparency, easy fabrication, low cost, and environmentally benign [25, 26]. The overall capacitance of conducting polymers (CPs) comes from the reversible redox reactions of *π*–*π* conjugated double bond present in the polymer complex, in addition to the double-layer charge storage mechanism of carbon present in the polymer matrix [27, 28]. Irrespective of their superficial potential, the industrialization of CPs-based capacitors is stalled by poor cycling stability owing to the bulk structures which constrain the consumption of material opening to electrolytes [29, 30]. Secondly, the adhesion of electrochemically polymerized film on planar current collectors has low adhesion, which causes high contact resistance and affects the electrochemical performance of the devices [31, 32]. Among CPs, polypyrrole (PPy), polyethylene dioxythiophene (PEDOT), and polyaniline (PANI) are the most studied CPs for microelectronics applications [33]. To achieve a stable device, constructing CPs with porous structure is one technique to overcome the instability problem of microelectrodes. Also, the electrochemical performance of CPs-based electrodes can be enhanced via hybridizing CPs with different carbon-based materials, for instance, graphene, CNTs [34, 35], and metal oxides such as MnO_2_ [36] and 2D materials such as MXene [37].

In CPs, PPy is an eye-catching pseudocapacitive material because of its comparatively high voltage window, low toxicity, easy polymerization route, high electrical conductivity, and comparatively low cost [38–41]. Conversely, the bulk structure of electrochemically polymerized PPy and its low cycling stability (< 50% after 1000 cycles) and low adhesion with planar current collectors limit its wide practicability in energy storage [25, 42]. The formation of charged nitrogen groups (polarons) on the PPy chain, as well as the migration of counterions from the electrolyte to PPy matrix, resulting the matrix to expand when it is oxidized. To maintain charge neutrality, counterions are pulled back to the electrolyte during reduction, causing the PPy matrix to shrink. The structural pulverization and activity loss of PPy is caused by the constant expansion and contraction [43, 44]. Second, adherence of the PPy film to the current collector is a problem. Because of its poor dedication stiff nature, the PPy film always pulls away from the current collector throughout the electrochemical reaction, resulting in high resistance and instability of devices. Another issue related to the electrochemical polymerization of PPy is the spreading of material on current collectors during long-term electrochemical polymerization, resulting in low accuracy in the areal capacitance of the device [45]. Currently, enormous efforts have been dedicated to constructing high-performance MSCs based on PPy; however, their cycling stability nor their energy density is adequate for the commercialization of PPy-based MSCs [46, 47].

Herein, we utilized a two-point strategy to fabricate high-performance PPy-based MSC by modifying the current collectors to overcome structural pulverization and enhance the adhesion of PPy film, followed by electrochemical co-deposition of PPy-CNT to grow highly porous nanostructures on rGO/Cr–Au current collectors. The resulting MSC with fine (negligible spreading of polymer) patterns achieved a high areal capacitance of 65.9 mF cm^−2^ at a current density of 0.1 mA cm^−2^ with a remarkable cycling performance, remaining 79% of the capacitance after 10,000 charge/discharge cycles at a high current density of 5 mA cm^−2^. Further, a current collector-free PPy-CNT@rGO flexible MSC was fabricated using a facile transfer approach on a flexible substrate yielding an areal capacitance of 70.25 mF cm^−2^ at 0.1 mA cm^−2^, and reserved 46% of the initial capacitance at a current density of 1.0 mA cm^−2^. Profiting the flexibility and high electrical conductivity, the flexible MSC is utilized as a micro-capacitive strain/pressure sensor with exceptional electromechanochemical properties.

## Experimental Section

### Assembly of rGO@Au Current Collector

**Fig. 1:**
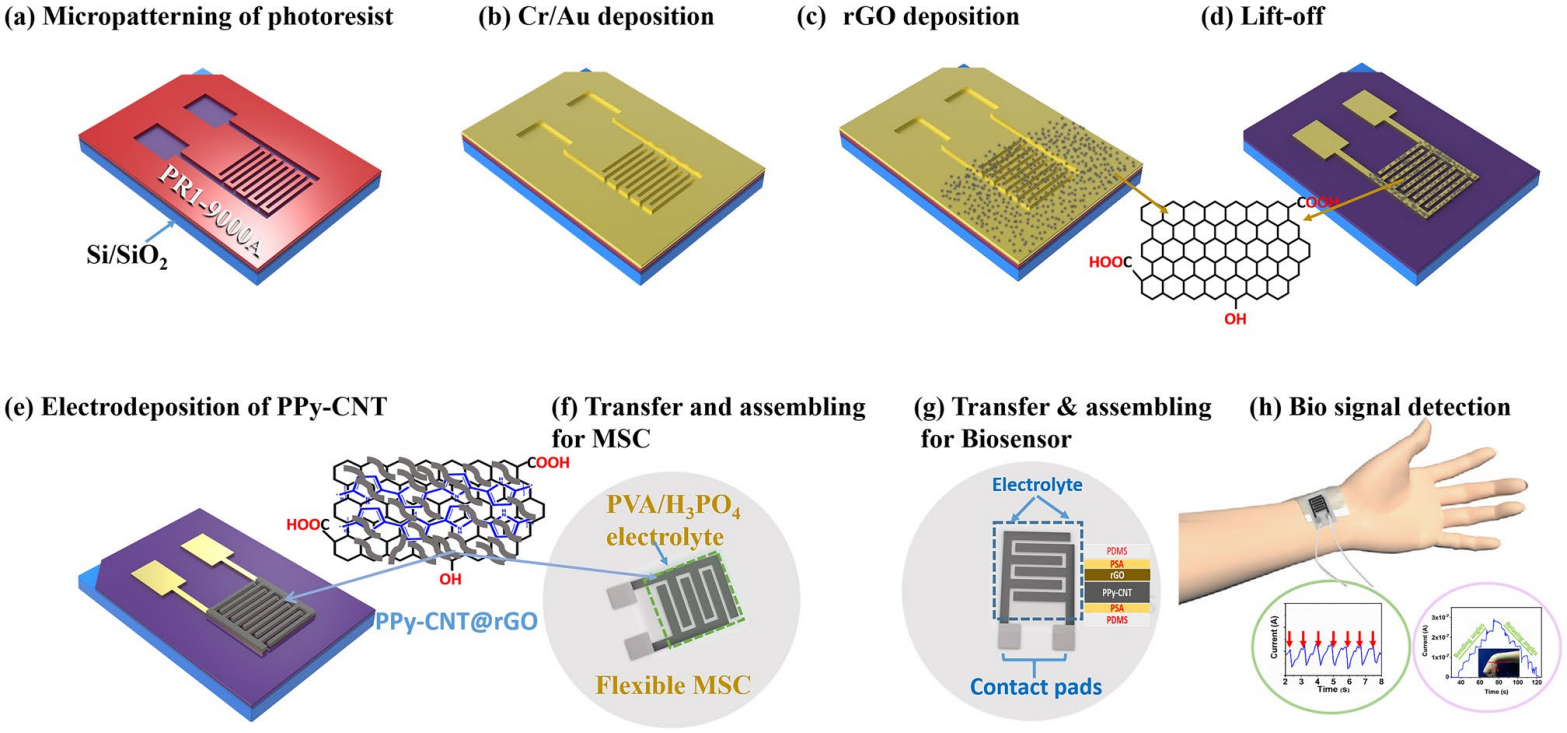
**a**-**h** A schematic of the microfabrication process of PPy-CNT@rGO MSCs and microsensors

The microfabrication process of PPy-CNT@rGO MSCs and microsensor is schematically shown in Fig. 1. A positive photoresist (PR1-9000A) was employed on a Si/SiO_2_ wafer, as reported previously [48]. In a nutshell, Si/SiO_2_ wafer (1.5 × 1 cm^2^) was cleaned using ultrasonication in isopropanol alcohol, ethanol acetone, and DI water sequentially. In the next step, the PR1-9000A layer was spin coated on the wafer (500 rpm, 10 s; 5000 rpm, 40 s). After exposing the sample to UV light through a photomask and developing it in RD-6 developer, the micropatterned photoresist was obtained. Thermal evaporation was then used to deposit a Cr/Au (5/50 nm) layer on the sample. The detailed dimension of interdigitated electrodes (length and width of fingers) is schematically displayed in Fig. S1. For multilayered graphene, 0.5 mL of 0.05 molar sodium sulfate solution was combined with GO solution (2 mg mL^−1^, 20 mL), and deposition was conducted on a sample by a three-electrode system at a constant current of 0.5 mA cm^−2^. Each deposition of 100 s was repeated to grow multilayered graphene.

### Fabrication of PPy-CNT@rGO MSC

Using a three-electrode setup, the PPy-CNT composite was electrochemically co-deposited on rGO@Au micropatterns. Pyrrole (120 µL) was mixed with deionized (DI) water (20 mL) by a continuous stirring process to make the electrolyte. Sulfuric acid was used to tune the pH of the solution to 4.0. An anionic surfactant sodium dodecyl sulfate (SDS, 10 mM) was added into the solution to reduce the deposition potential and improve the miscibility of PPy, and the solution was stirred for 30 min until a clear solution without suspended particles was achieved. Carbon nanotubes (0.002 g) with an average length of 1–2 µm were then sonicated into the aforesaid solution until a black suspension was obtained. The cyclic voltammetry (CV) deposition was accomplished at a scan rate of 2 mV s^−1^ for 7 cycles (180 s) under the voltage range of 0–0.75 V. After deposition, the sample was washed with DI water to remove the unreacted material and kept in an oven at 60 °C for overnight to get it fully dried. For fabrication of the PPy-CNT@rGO MSC, PVA/H_3_PO_4_ gel electrolyte was employed for further electrochemical characterizations. For PPy@Au MSCs, the electrolyte solution was prepared by the same way without the addition of CNTs, and the deposition was performed on Cr/Au micropatterns.

### Fabrication of PPy-CNT@rGO Flexible MSC/Pressure Sensor

For the fabrication of interdigitated flexible microelectrodes, the prepared PPy-CNT@rGO micropatterns on Au/Si/SiO_2_ were transferred on a cured flexible PDMS substrate. The substrate was treated with dipropylene as epoxy and after curing, the PPy-CNT@rGO microelectrodes on Au/Si/SiO_2_ were placed on the PDMS substrate. The sandwiched microelectrodes were placed on a preheated plate at 40 °C under pressure. After 15 min, the PDMS was peeled from the silicon substrate. The micropatterns were completely transferred on the PDMS substrate without any displacement of the channels. For testing the electrochemical/electromechanochemical properties, the microelectrodes were coated with desired electrolytes. After drying, the MSC/microsensor was assembled by covering the microelectrodes with another thin PDMS membrane except for the contact pads.

### Materials and Characterizations

A field emission scanning electron microscope (FE-SEM, JSM-7100F) and an optical microscope (Sunny Optics, 50/100 objective) are used to examine the surface morphology and structure of samples. The submicron features of microelectrodes are studied using a transmission electron microscope (TEM-1400-P). Raman spectra of samples are collected using a Renishaw RM-1000 laser Raman microscope instrument. The microelectrodes thickness is measured with a step surface profiler (ET4000A). BET surface area is obtained by Horiba SA-9600. A VG Multilab 2000 is utilized to record X-ray photoelectron spectroscope (XPS) spectra of samples. The CHI-760 and Autolab PGSTAT302N electrochemical workstations are employed to carry out the electrochemical testing.

## Results and Discussion

### Morphological and Structural Characterizations

**Fig. 2:**
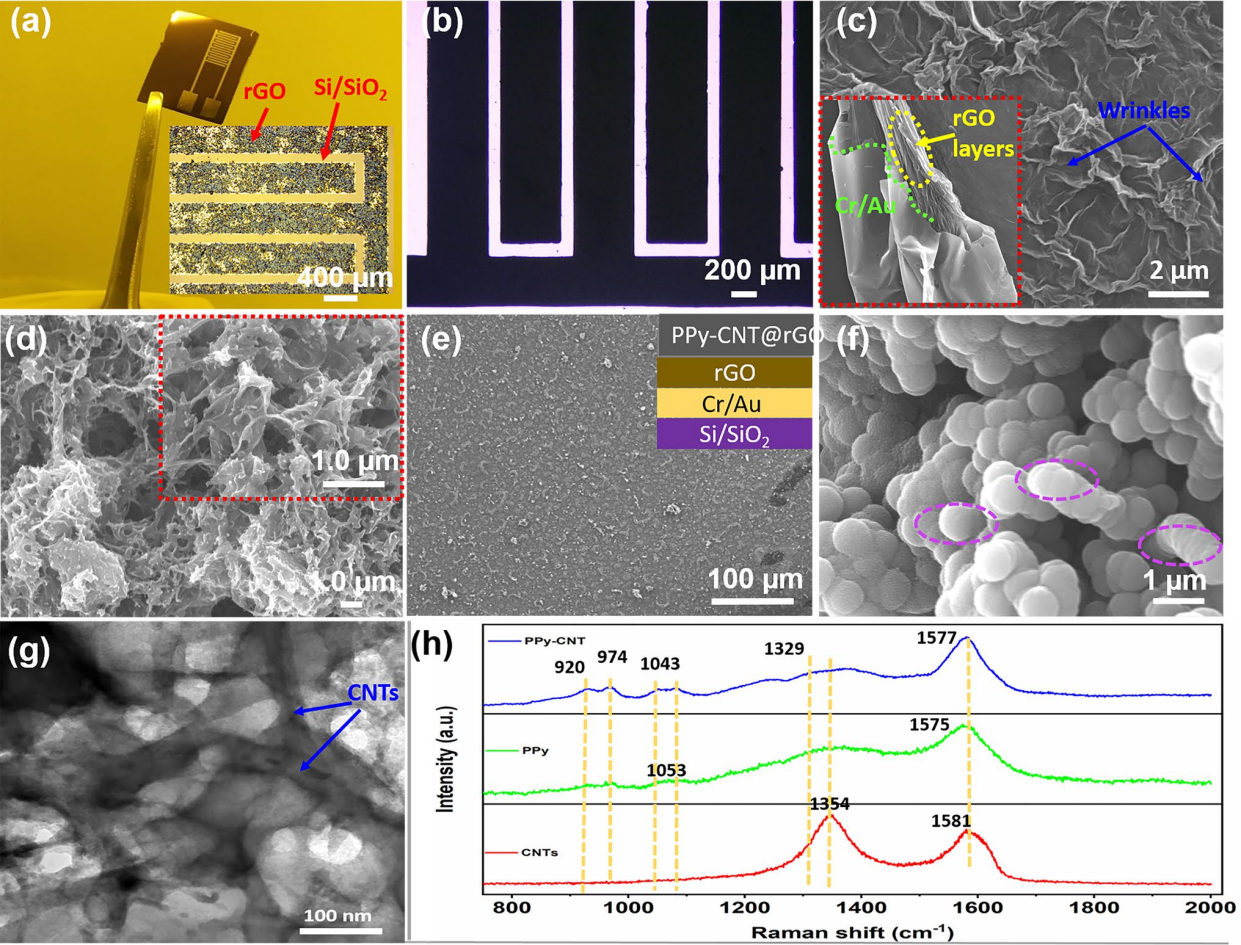
Structural characterization results. **a** Photographic image of interdigital Cr/Au micropatterns, the inset shows the microscopic image of rGO@Au microelectrodes. **b** Optical image of PPy-CNT@rGO MSC. **c** SEM image of electrodeposited rGO on micropatterns. **d** SEM image of PPy-CNT on rGO by short-time deposition. **e**, **f** Low- and high-resolution SEM images of PPy-CNT composite electrochemically deposited on rGO@Au micropattern. **g** TEM image of PPy-CNT. **h** Raman spectra of CNTs, PPy and PPy-CNT microelectrodes

The digital photograph of Cr/Au micro-current collectors on a Si/SiO_2_ wafer is displayed in Fig. 2a, whereas the inset shows the optical microscopic image of electrodeposited rGO uniformly coated on Cr/Au micropatterns. The advantage of post-lift-off is fine micropatterning of rGO@Cr/Au without any graphene sheets within the interspace of microelectrodes. The post-lift-off deposition resolved the problems of spreading of rGO in finger interspaces which leads to short circuits, and the spreading of polymer during the second deposition. Also, the electrodeposited layered graphene is highly stable, and a fine pattern even after lift-off in acetone is obtained.

**Fig. 3:**
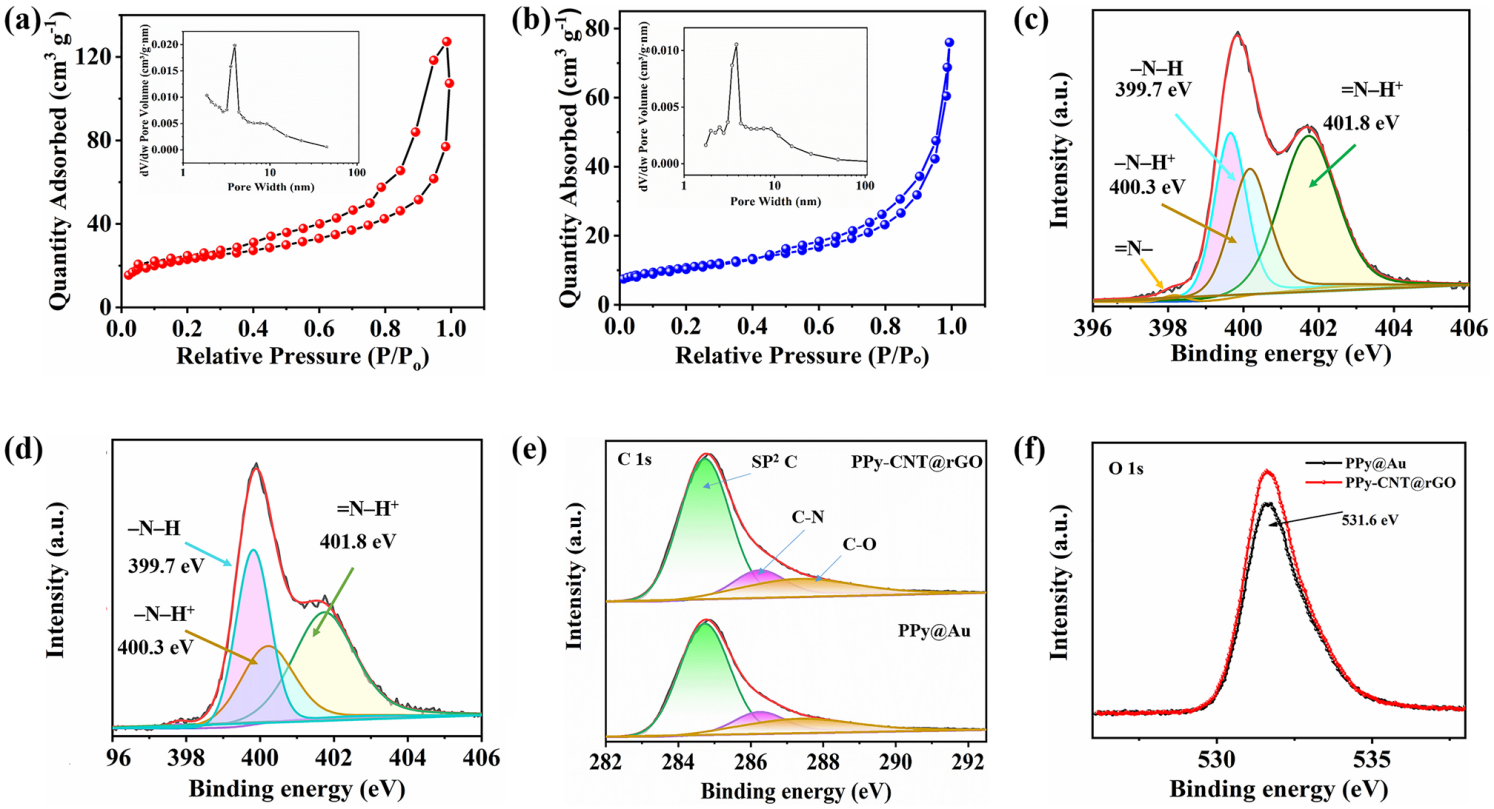
**a, b** The nitrogen adsorption–desorption isotherms of PPy-CNT@rGO and pristine PPy. The insets show the corresponding pore size distributions. **c, d** XPS spectra of N1s core level of PPy-CNT@rGO nanocomposite and PPy@Au, respectively. **e, f** Deconvolution of the C 1*s* and O 1*s* spectra of PPy-CNT@rGO and PPy@Au

The detailed microscopic and SEM images of rGO on Cr/Au patterns are displayed in Fig. S2. Figure 2b shows the optical microscopic image of the PPy-CNT composite electrochemically deposited on rGO@Au micropatterns. For controlled deposition (at low potential and short time), the polymer-CNT composite is precisely deposited on the rGO@Cr/Au microelectrodes resulting in the development of a very fine micropattern without the spreading of polymer within the interspace between the fingers of electrodes. The electrochemically deposited rGO on Cr/Au micropatterns has surface step layers and fringes (Fig. 2c) which are suitable for the deep penetration of electrolyte ions, and also useful for the growth of highly stable PPy-CNT composite with low contact resistance and strong surface adhesion. The PPy and PPy-CNT electrolytes prepared for electrochemical deposition and corresponding PPy and PPy-CNT microelectrodes are displayed in Fig. S3a–d. Figure 2d shows the SEM image of the PPy-CNT@rGO aerogel-type structure of microelectrode for a short polymerization time (10 s). The CNTs along with the polymer matrix were deposited on the rGO. Figure 2e shows the low-resolution SEM image of PPy-CNT@rGO microelectrodes. Figure 2f displays the polymer-coated CNTs with highly porous structure and enhanced surface area, offering high specific surface area and the ability to compensate for large volumetric changes during electrochemical performance. Further assimilation of CNTs in PPy matrix is displayed in Fig. S4. TEM images of co-electrodeposited PPy-CNT and pristine PPy are displayed in Figs. 2g and S5. The CNTs are fastened within the polymer matrix and facilitate the aligned growth of the polymer. The decrease in the interspace distance with pyrrole polymerization of 180 s is displayed in Fig. S6a-d. The interspace distance decreased to 25 µm from 180 µm with a deposition time of 3 min, which results in an increase of overall 19% area of microelectrodes and low accuracy in energy and power of the device. The thickness of the electrodes is measured with a step surface profiler. The average thicknesses of the PPy@Au, PPy@rGO/Au, and PPy-CNT@rGO/Au microelectrodes are found to be 3.2, 2.8, and 4.2 µm, respectively (Fig. S7). Figure 2h demonstrates the Raman spectra of CNT, PPy, and PPy-CNT. The CNT reveals two distinctive peaks at 1354 and 1581 cm^−1^, tailored to disorder mode (D-band) and tangential mode (G-band), separately. The distinct peaks of primeval PPy and PPy-CNT at 920 and 974 cm^−1^ resemble to C-H deformation plane and polaron arrangements of pyrrole rings, respectively. Similarly, the peaks at 1053 and 1043 cm^−1^ match well with C-H in-plane deformation of PPy and PPy-CNT, respectively. PPy-CNT shows an additional peak positioned at 1329 cm^−1^, probably due to C–C stretching. In addition, for PPy-CNT, the C = C vibration bond shifts to 1577 cm^−1^ (for PPy, C=C vibration peak is located at 1575 cm^−1^). Associated with CNT, the D-band is not perceived and the G-band is stretched for PPy-CNT, signifying the PPy coating on the surface of CNT. To obtain the quantitative data on the specific surface area (SSA) and pore size/volume, we performed BET analysis of PPy-CNT@rGO and pristine PPy (Fig. 3a, b). The results demonstrate that PPy-CNT@rGO possesses a high BET surface area of 81.25 m^2^ g^−1^ with a pore volume of 0.183 cm^3^ g^−1^. The pristine PPy possessed a low BET surface area of 37.4 m^2^ g^−1^ with a pore volume of 0.108 cm^3^ g^−1^ which is almost half of the PPy-CNT@rGO. The enhanced SSA and appropriate pore volume (analogous to the volume of electrolyte ions) are beneficial to the large area permeation of electrolytes for high electrochemical performance.

High-resolution X-ray photoelectron spectroscopy (XPS) investigation was executed to reveal the promising bonding, comprising of oxidation states of PPy-CNT@rGO nanocomposite and PPy@Au besides their estimated composition. The XPS spectra of core level N1s of PPy-CNT@rGO and PPy@Au are displayed in Fig. 3c, d, respectively. The sharp peak located at 399.7 eV is attributed to the (–N–H) charge-free nitrogen present in the PPy ring. The second merged peak positioned at 400.3 eV corresponds to the (–N–H^+^) state. The third peak with a little high intensity at a binding energy of 401.8 eV is attributed to =N–H^+^, which might come from the existence of bipolaron charge haulers inside the PPy matrix.

However, for the N1s spectrum in PPy-CNT@rGO, the converse phenomenon is anticipated because, in N atoms, the electron density upswings over the establishment of the hydrogen bonds. This characteristic future is observed in Fig. 3d where a negative shift of the N 1s spectrum designates the electron donation within the oxygen functionalities of carbon, and is responsible for the electron displacements in the locality of the nitrogen atoms. The atomic percentages of carbon and oxygen atoms in PPy-CNT@rGO are higher than those of PPy@Au due to the addition of CNTs and some oxygen functional groups in graphene (Fig. 3e). Similarly, in O 1*s* XPS spectra, the intensity of oxygen peak in PPy-CNT@rGO is higher than that of PPy@Au (Fig. 3f).

### Electrochemical Performances MSCs

**Fig. 4:**
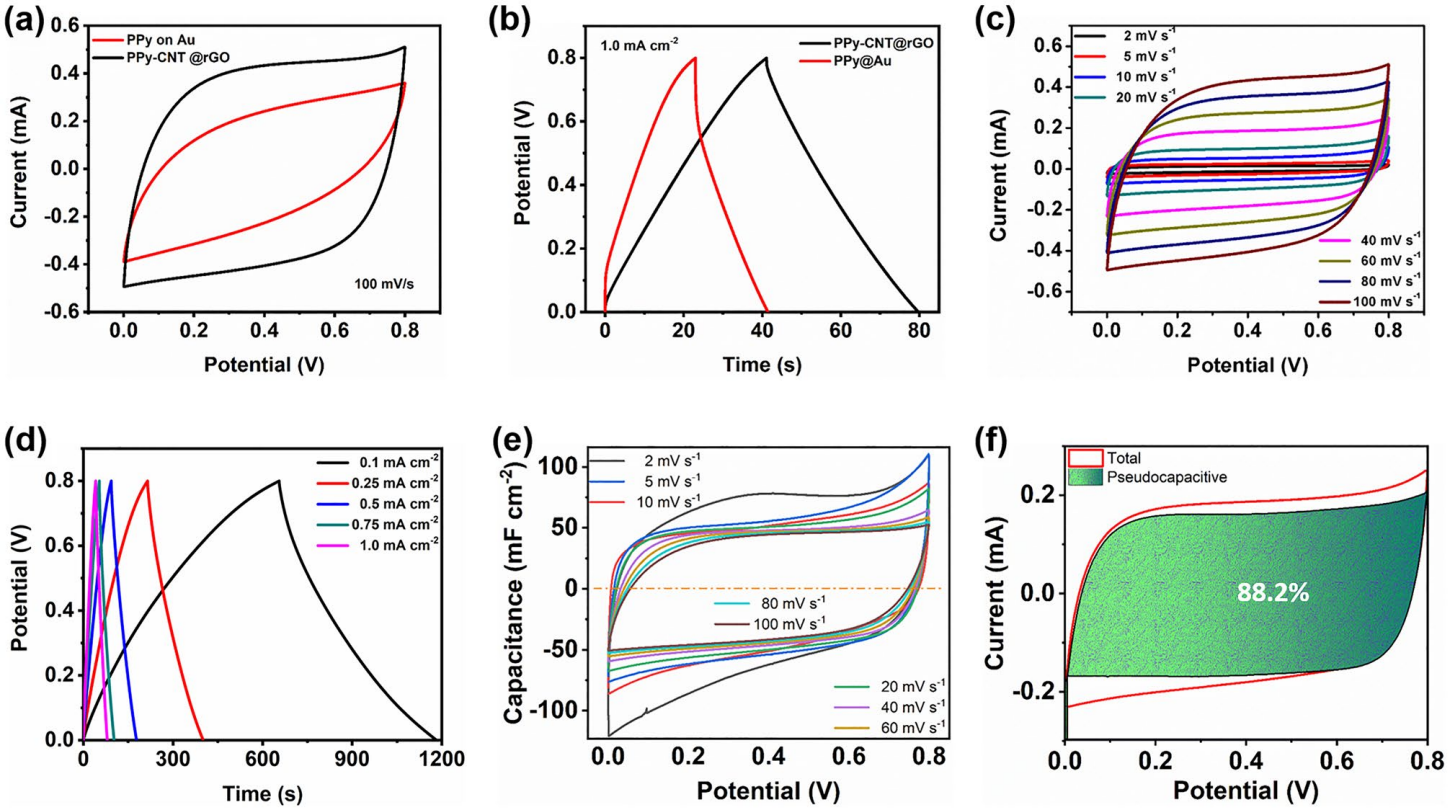
**a** CV curves of PPy-CNT@rGO and PPy@Au MSCs at a scan rate of 100 mV s^−1^. **b** GCD curves of PPy-CNT@rGO and PPy@Au MSCs at a current density of 1 mA cm^−2^. **c** CV curves of PPy-CNT@rGO MSC at different scan rates from 2 to 100 mV s^−1^. **d** GCD curves of PPy-CNT@rGO MSC at different current densities from 0.1 to 1.0 mA cm^−2^. **e** Areal capacitance at different scan rates with the potential range from 0 to 0.8 V. **f** Capacitance separation analysis of PPy-CNT@rGO MSC at a scan rate of 5 mV s^−1^

To evaluate the electrochemical activity of PPy-CNT@rGO quasi-solid-state MSC, the electrochemical experiments were executed by a two-electrode system using PVA/H_3_PO_4_ gel electrolyte. Figure 4a, b displays the cyclic voltammetry (CV) curves (scan rate of 100 mV s^−1^) and galvanostatic charge/discharge (GCD) curves (at a current density of 1 mA cm^−2^) of PPy-CNT@rGO and PPy@Au MSCs, respectively. The CV curve of PPy-CNT@rGO shows a quasi-rectangular shape even at such an elevated scan rate, whereas, for PPy@Au MSC, due to its high ion transport resistance, low porosity, and low electrical conductivity, the shape of CV curve is not rectangular, showing the nonlinear relationship of applied voltage and output current. Also, it is significant from Fig. 4a that the capacitance of PPy-CNT@rGO MSC is higher than that of PPy@Au MSC. The reason is the high surface area with the enhanced porous structure of PPy-CNT composite deposited on rGO, which has high electrical conductivity and good adhesion to the substrate for delivering current rapidly. The similar results can be drawn from the GCD curves of both the MSCs. The discharge time of PPy-CNT@rGO MSC is longer than that of PPy@Au MSC with a very small *iR* drop compared with that of PPy@Au MSC (Fig. 4b). To further evaluate the electrochemical performance of PPy-CNT@rGO MSC, the CV curves at diverse scan rates (2–100 mV s^−1^) were taken and displayed in Fig. 4c. The representative rectangular nature of CV curves even at 100 mV s^−1^ and rectilinear variation in current with scan rate shows a typical pseudocapacitive mechanism, excellent rate proficiency, and reversibility of PPy-CNT@rGO MSC. GCD is the most versatile method for the actual evaluation of electrochemical capacitive performance. GCD tests were performed at different current densities (from 0.1 to 1 mA cm^−2^) within a voltage window of 0–1 V (Fig. 4d). The CV curves at higher scan rate (0.2 to 0.5 V s^−1^) and GCD curves at higher current densities (2 to 10 mA cm^−2^) are displayed in Fig. S8. On increasing the scan rate of PPy-CNT@rGO MSC from 200 to 500 mV s^−1^, the shape of CV is still quasi-rectangular, showing the high rate performance of PPy-CNT@rGO MSC (Fig. S8a). Further, on increasing the current density from 2 to 10 mA cm^−2^, the value of *iR* drop is increased from 0.05 to 0.15 V, hence maintaining the triangular shape of GCD curves (Fig. S8b).

For PPy@Au MSC, at a low scan rate, the shape of the CV is quasi-rectangular which becomes skewed as the scan rate increases, resulting in the poor rate performance of the PPy@Au device. The areal capacitances of PPy@Au MSC calculated from GCD (at 0.1 mA cm^−2^) and CV (scan rate of 2 mV s^−1^) are 37.7 and 37.2 mF cm^−2^, respectively. For comparison, the CV (at various scan rates) and GCD (at different current densities) curves of PPy@Au MSC are illustrated in Fig. S9. The area-specific capacitance of PPy-CNT@rGO MSC at a current density of 0.1 mA cm^−2^ is calculated to be 65.9 mF cm^−2^, which is the highest among on-chip planar PPy-based MSCs. Also from the CV curves at a scan rate of 2 mV s^−1^, the areal capacitance is calculated to be 64.8 mF cm^−2^ which matches well with the capacitance calculated from the GCD curves.

As PPy stores charge by means of reversible electrochemical doping, that is categorized by a slightly quadrilateral cyclic voltammogram which will reveal a set of reversible redox peaks. This procedure includes either the oxidation of the CP backbone with the simultaneous addition of an anion through the electrolyte (*p*-doping), or the removal of cation from polymer back to the electrolyte, producing unconstrained charge carriers alongside the polymer chains. The CV profiles of PPy-CNT@rGO electrode at a comparatively high scan rate look almost rectangular. However, at the low scan rate (2 mV s^−1^), there are sharp redox peaks at the edges and a wide peak around 0.35 V (Fig. 4e). Based on Fig. 4c, the capacitance contributed by surface capacitive effect and diffusion-controlled contribution in overall capacitance is calculated by Eq. (1):

1 $$i\left( v \right) = k_{1} v + k_{2} v^{\frac{1}{2}}$$

where *i*(*v*) represents current under a specific voltage, *k*_*1*_ and *k*_*2*_ are two constants, and ${k}_{1}v$ and ${k}_{2}{v}^\frac{1}{2}$ denote the currents contributed by surface capacitive effect and diffusion-controlled processes, respectively.

By employing Eq. (1), the capacitive and diffusion-controlled contributions are calculated to be 75%, 78%, 82%, and 88% at scan rates of 5, 10, 20, and 40 mV s^−1^, respectively (Figs. 4f, S10d).

**Fig. 5:**
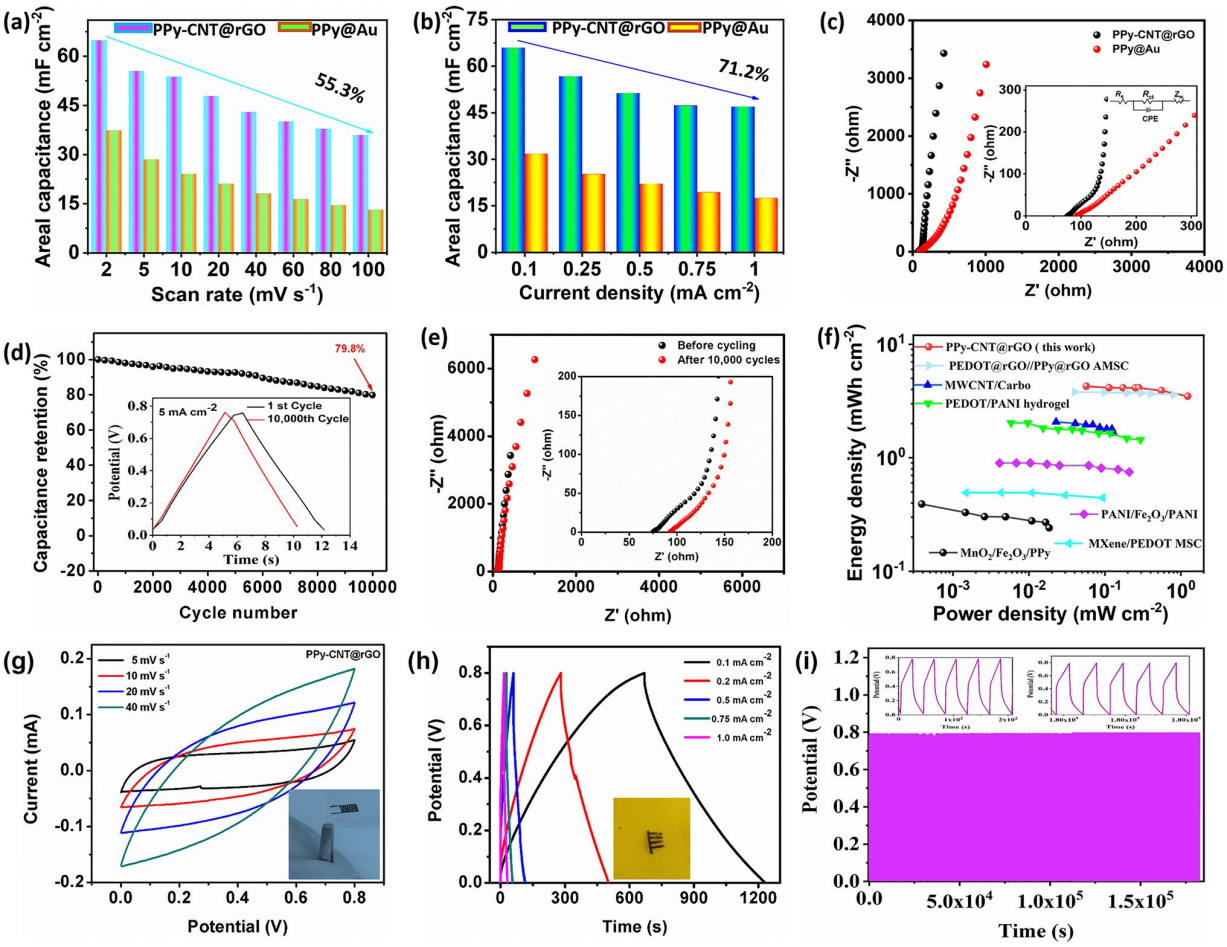
**a, b** Areal capacitance as a function of scan rate and current density for PPy@Au and PPy-CNT@rGO MSCs. **c** EIS curves of the fabricated solid-state MSCs, and the inset shows its zoom view at the high-frequency region. **d** Cycling stability of MSC measured at a very high current density of 5 mA cm^−2^ within a voltage window of 0.8 V. **e** EIS curves of the fabricated solid-state MSC before and after 10,000 cycles. The inset shows its zoom view at high-frequency region. **f** Ragone plot revealing areal energy density *vs.* power density of PPy-CNT@rGO MSC in comparison with recently reported CPs-based MSCs [49–52]. **g, h, i** CV, GCD and cycling stability curves of current collector-free PPy-CNT@rGO flexible MSC

To calculate the rate capabilities of the fabricated MSC, the relationship of areal capacitance with scan rate and current density is measured and displayed in Fig. 5a, b. On increasing the scan rate, the areal capacitance decreases and remains almost linear (Fig. 5a). At a high scan rate of 100 mV s^−1^, the PPy-CNT@rGO MSC retains 55.3% of its initial capacitance at 200 mV s^−1^ (compared with ~ 30% of PPy@Au), showing the device’s good rate capability and excellent reversibility. Figure 5b displays the effect of areal capacitance on current density. As the value of applied current increases, there is a decline in the capacitance of MSC. When the current density is increased from 0.1 to 1 mA cm^−2^, the areal capacitance is decreased from 65.9 to 47 mF cm^−2^, retaining 71.2% of the capacitance (compared with 51% for PPy@Au), displaying the good rate capability of PPy-CNT@rGO MSC.

Figure 5c demonstrates the complex Nyquist plots (frequency range of 10^–2^ to 10^5^ Hz) of the PPy-CNT@rGO and PPy@Au MSCs. The consolidated resistance of the MSCs is deliberated by the addition of effective series resistance (ESR) and equivalent distributed resistance (EDR) in the high-frequency constituency of plot, which is about 150 Ω for PPy-CNT@rGO and 246 Ω for PPy@Au MSC, respectively. This is due to the fact that embedded CNTs improve electrical conductivity and lower the ohmic resistance. Besides, the enhanced surface area of the PPy-CNT composite enables the electrolyte contact with the polymer CNTs matrix to form a double layer and ultimately in the low-frequency region, the EIS plot of PPy-CNT@rGO is nearly parallel to the y-axis (imaginary axis) which validates the low ions diffusion resistance and perfect charge storage mechanism of the MSC. However, the deflection of the plot in the low-frequency region for PPy@Au MSC away from the imaginary axis is due to the high ion diffusion resistance in the bulk of PPy deposited on Au. One of the utmost essential electrochemical tests for CPs-based MSCs is the cycling stability measurement. PPy-CNT@rGO MSC was cycled for 10,000 charge/discharge successive cycles at 5 mA cm^−2^ within a voltage window from 0 to 0.8 V (Fig. 5d). The quasi-solid-state MSC showed an excellent cycling performance, retaining 79.8% of the initial capacitance after 10,000 GCD cycles, which is higher than previously reported rGO and PPy-based MSCs [49, 53–55]. For comparison, the PPy@Au MSC is cycled under the same conditions and the results are displayed in Fig. S11. The excellent cycling performance of PPy-CNT@rGO MSC is due to two factors. The first one is the flexible nature of rGO current collectors which will accommodate the structural pulverization of the polymer through uninterrupted charge/discharge cycles due to the exchange of polarons between the electrolyte and active material. Secondly, the addition of CNTs modifies the structure from bulk to porous of the PPy matrix which is favorable to accommodate large volumetric changes and also enhance the surface area to boost the electrochemical performance. The highly stable rGO with many wrinkles on its surface increases the adhesion of the polymer composite with current collectors, ensuring low contact resistance and superior electrical conductivity. The coulombic efficiency of the MSC is calculated by using Eq. (2).

2 $$\eta = \frac{{\Delta t_{{\text{d}}} }}{{\Delta t_{{\text{c}}} }} \times 100$$

where ${\Delta t}_{\mathrm{d}}$ and ${\Delta t}_{\mathrm{c}}$ represent the discharge and charge time, respectively. Figure S12 shows that the coulombic efficiency of a PPy-CNT@rGO MSC increases from 80.7% to 98.9% as the current density increases from 0.1 to 1.0 mA cm^−2^ with a little iR drop, which confirms the excellent charge/discharge reversibility of the MSC, even with a high areal capacitance of 65.9 mF cm^−2^. The PPy@Au MSC could attain a Coulombic efficiency of 75% and 94% at a current density of 0.1 and 1.0 mA cm^−2^, respectively.

The electrochemical impedance spectroscopy (EIS) tests were performed before and after cycling for PPy-CNT@rGO MSC (Fig. 5e). The compared charge transfer resistances of PPy-CNT@rGO before and after cycling show a small increase in the resistance of the device after 10,000 GCD cycles. We attribute this to the porous structure of PPy-CNT composite and strong adhesion with current collectors, which are advantageous to the long-span cycling stability of the MSC. Contrary to this, the PPy@Au MSC has a low cycling performance of 49% after 10,000 charge/discharge cycles due to the low adhesion and structural pulverization of the PPy matrix. The energy and power densities of PPy-CNT@rGO and PPy@Au MSCs are calculated and sketched in Fig. 5f. The fabricated PPy-CNT@rGO MSC delivered the maximal areal energy density of 5.8 µWh cm^−2^ and the maximum power density of 0.4 mW cm^−2^ at an energy density of 4.17 µWh cm^−2^, which is higher than those of previously reported on-chip planar polymer-based MSCs, as displayed in Ragone plot (Fig. 5f). The PPy@Au MSCs achieved the maximum energy density of 2.7 µWh cm^−2^ and the maximum power density of 0.348 mW cm^−2^ at an energy density of 1.45 µWh cm^−2^. Table S1 shows the comparative electrochemical performances of the recently reported conducting polymers such as PPy, PEDOT, and PANI and their composites-based MSCs in details.

For practical applications in electronic devices/systems, the microdevices are usually coupled in series or parallel to get desired energy and power. Herein, we demonstrate the practicability of MSCs by connecting them in series and parallel. By connecting three MSCs in a series configuration, a voltage window up to 2.4 V is achieved without the prominent loss in capacity (Fig. S13a, b). Also, the CV and GCD curves of single and three MSCs in series show evidence of the compatibility of MSCs in electronic circuits. By joining three MSCs in parallel, a threefold discharge time beyond a single device is attained at a constant potential of 0.8 V.

Current collectors play a vital role in the performance of an energy storage device. Concerning MSCs, material design, processing, and current collectors’ surface properties can impact considerable variations in energy density, power output, cycling stability, and other crucial performance constraints. However, the metallic current collectors hinder the plasticity of the devices to make flexible electronics. Furthermore, a current collector-free flexible MSC is also achieved by transferring the PPy-CNT@rGO microelectrodes on a flexible substrate. Figure S14 shows the digital photographs of the PPy-CNT@rGO flexible MSC. It is clear that after transferring, the micropatterns are still stable and maintain their shapes without any deformation and short circuit of microelectrodes. Figure 5g shows the CV curves of PPy-CNT@rGO flexible MSC at the diverse scan rates from 5 to 40 mV s^−1^. At a low scan rate, the shape of the CV is rectangular, showing the typical pseudocapacitance and high electrical conductivity even without metal current collectors. As more active material is exposed to electrolyte after transferring on a flexible substrate, the areal capacitance of the MSC is increased. However, the rate performance is affected because the flow of electrical charges from polymer matrix at a high scan rate is less than that of Au current collectors. The areal capacitance from GCD curves at a current density of 0.1 mA cm^−2^ is calculated as high as 70.25 mF cm^−2^ (Fig. 5h). Further, areal capacitances are plotted against current densities to show the rate performance of flexible PPy-CNT@rGO MSC (Fig. S15a). At a current density of 1.0 mA cm^−2^, the MSC retained 46% of the initial capacitance at 0.1 mA cm^−2^, higher than or comparable to those of many carbons based on previously reported MSCs [56, 57]. To know the long-term stability of metal-free PPy-CNT@rGO MSC, repeated charge/discharge cycles at a current density of 1 mA cm^−2^ are performed for about 10,000 cycles within a voltage window from 0 to 0.8 V. The metal-free flexible MSC delivers an excellent cycling performance, retaining 86% and 76% of the initial capacitance after 5000 and 10,000 cycles, respectively. Figure 5i shows the GCD profiles of the MSC with the inset showing the first and last five cycles. The rGO facilitates the power output in the absence of metal current collectors in addition to preventing structural pulverization of the polymer matrix. The in situ EIS is utilized for simultaneous measurements of the impedance spectra with charge/discharge curves by galvanostatic control (Fig. S15b). The Rs and charge transfer resistance (*R*_ct_) after the first cycle obtained from the simulation of the Nyquist plot are 121.2 and 0.8 Ω, respectively. During 10,000 charge/discharge cycles, the Rs increases to 122.5 Ω, whereas the Rct increased to 85.2 Ω. The increased *R*_ct_ suggests that the PPy-CNT@rGO flexible microelectrodes suffer ions diffusion resistance after such long cycling, resulting in a 24% capacitance decay after 10,000 cycles. The high electrical conductivity and flexibility of the current collector-free MSC extend its utilization toward flexible electronic devices.

### MSC as a Capacitive Micro-Strain Sensor

Flexible pressure/strain sensors are required for numerous applications, for instance, human organ motion sensing, health monitoring, robotics, and human–machine interaction. For such types of applications, the sensors must be lightweight, highly compatible with electronic devices, soft, and mechanically durable for long-term use. Strain sensors are performed as transducers, which transform an applied mechanical distortion of strain into electronic signals such as a variation in resistance, capacitance, current, or voltage, depending on the type of sensing mechanism utilized in the sensor. Capacitive pressure sensors in recent era are constructed similar to the architecture of thin film capacitor, which contains two conducting electrodes stacked together with a dielectric layer/electrolyte between electrode plates [58–60]. The sandwich-like configuration is applicable to most of the active materials as well as cost-effective for mass production. However, from practical aspects, it suffers from obvious drawbacks, such as possibility of short circuit with applied pressure, unwanted dislocation of electrodes, and utilization of more active materials. The second limitation of stacked design is to control the small distance (50–100 µm) between electrodes which normally decides the response time of the sensor. Compared with conventional sandwich-like design, the planar interdigital design of electrodes has more advantages despite their complex synthesis process. The prominent advantage of in-plane interdigital design is the narrow interspace for the dielectric layer between the electronically isolated electrode fingers obtained by lithography and transfer method. The finger electrodes with micrometer size are easy to produce tensile strain under tiny stress and give strong signals in the form of current, voltage, or resistance variations. Similarly, the micropores in active materials can instantaneously diffuse the electrolyte ions into the interspaces within the electrodes when a certain pressure is applied and intercalate the ions back to the pores while releasing the pressure, giving rise to the variation in electronic signals with faster response. We utilized PPy-CNT@rGO MSC as a micro-strain/pressure sensor on an elastic substrate to record applied stress and human organs motion, which demands numerous patterned designs based on the purpose of identifying mechanical deformations, for instance, bending, stretching, and pulse/beat detection. The microelectrodes with low deposition time and less thickness compared with MSC were transferred on a flexible PDMS elastomer.

**Fig. 6:**
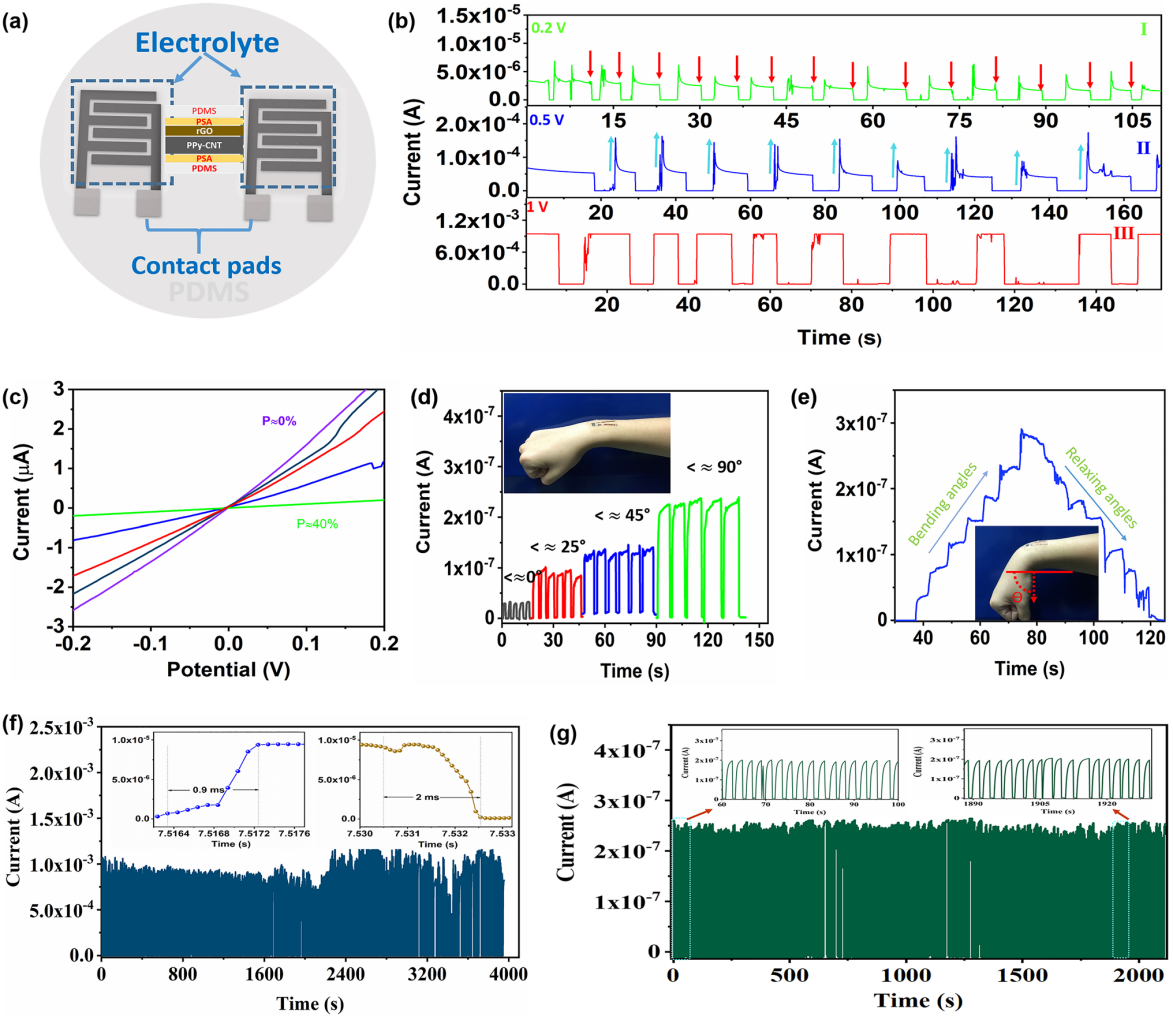
Electromechanochemical performance of the MSC-derived MSS. **a** Schematic diagram of flexible MSS. **b** Variation in current with change in applied stress at different applied voltages for flexible MSS. **c** Current voltage curves of micro-strain sensor under various applied stresses from 0 to 40% within voltage limits of − 0.2 to 0.2 V. **d** The response and decay curves at different angles. **e** The relative change in capacitive current from the gradual wrist bending and relaxing over five bending angles of 10°, 25°, 45°, 75°, and 90°, respectively. **f** Mechanical test results of cyclic durability for 2500 stress/release cycles at an applied voltage of 1.0 V and a maximum stress of 50 kPa. The inset shows the response time (rise and decay time). **g** Cyclic durability for 800 bending/release cycles for PSA/PVA microsensor with the maximum bending angle of 90°. The inset shows the first and last few cycles

The cured PDMS was treated with two different electrolytes depending on the desired performance. The MSC with quasi-solid-state electrolyte (PVA/LiCl) gives higher initial output currents at different applied potentials. Figure 6a shows the schematic of the fabrication of flexible micro-strain sensor. The transferred microelectrodes are covered with another thin PDMS slab. One side coated with electrolyte is directed toward to microelectrodes. The applied stress in this case lowers the value of current to the minimum value at the maximum applied stress. At different applied voltages, the sensor gives different output currents due the different amounts of charge stored in the device. By applying variable stresses, the current starts decreasing and finally reaching zero at applied stress of some level. The pores inside the microelectrodes shrink under applied force and lower the output current to its lowest value. Figure 6b(I–III) displays the current *vs*. time profiles of microsensor at 0.2, 0.5, and 1 V, respectively. At 0.2 V, the initial output current obtained by sensor is about 3 µA. With the applied stress, the current reduces and finally approaches the minimum value. On releasing, the sensor again achieves almost the same initial current. Multiple repetitions were made with specific time interval of press and release, and DC current-like waves are obtained with quick response (Fig. 6b–I). Similarly, Fig. 6b(II–III) displays the similar behaviors at different applied voltages of 0.5 and 1 V, respectively, with different output signals. To verify the effect of applied pressure on output current signals, we performed linear scan voltammetry (LSV) at relax conditions and under different stresses up to 40% (4 kPa). At relax state, e.g., at the applied stress of 0 Pa, the sensor displayed a maximum current of 3 µA around 0.2 V, whereas, at the applied stress of 4 kPa, the value of current is reduced to 0.1 µA, which approaches to zero at comparatively higher strain. The other values of current at different stress of 10%, 20%, and 30% are displayed in Fig. 6c. Similarly, the CV curves of the assembled flexible MSC at a scan rate of 40 mV s^−1^ under different applied stress from 0 Pa to 10 kPa verify the LSV results. The CV curves and corresponding digital photographs of the pressure gauge displaying the different amounts of applied stress are displayed in Fig. S16.

To demonstrate the sensitivity of sensor, we choose the wearable applications, where the motions of bending, and relaxing of a wrist are observed. The microsensor is placed on the wrist joint (between radius and ulna) and at different bending angles, the electrical signals were recorded (Fig. 6d). After each bending at specific angle, the wrist is brought back to initial position, and the rise and decay currents are recorded with fast response. Furthermore, the continuous strain steps without relaxing wrist are applied to observe the practicability of the microsensor. The wrist bending gestures consist of six angles starting at relax (horizontal) position is 0°, and then, it is bent to 10°, 25°, 45°, 75°, and 90°, respectively. The current steps increase linearly as the bending angle steps increase. The similar variations in the current are observed while relaxing the wrist back to the normal position. However, due to uncontrolled movement, there are some dissimilarities in current profile during unloading strain (Fig. 6e). In order to test the electromechanical stability, the sensor was subjected to continuous stress and relax repetitions for more than one hour (2500 stress/relax cycles). The sensor presented a stable response within 2500 consecutive cycles (Fig. 6f). One of the most significant parameters of a pressure sensor is the response and recovery time. The interdigital strain sensor displayed a low response and recovery time of 0.9 and 2 ms, respectively (the inset in Fig. 6f), much shorter than those of recently reported traditional capacitive strain sensors. Similarly, to further verify the current retention rate and stability, the PSA/PVA-based sensor is cycled for more than 800 bending cycles. The sensor displayed stable current time profiles with almost 100% current retention. The configuration and response/recovery time of our integrated microsensor and recently reported traditional capacitive strain sensors are compared in Table S2. These results demonstrated that the interdigital flexible micro-strain sensor is capable of sensing different ranges of stress (from less than 1 Pa to 10 kPa) with high sensitivity, short response/relaxation time, and small size compatible with the latest miniaturized electronic devices.

**Fig. 7:**
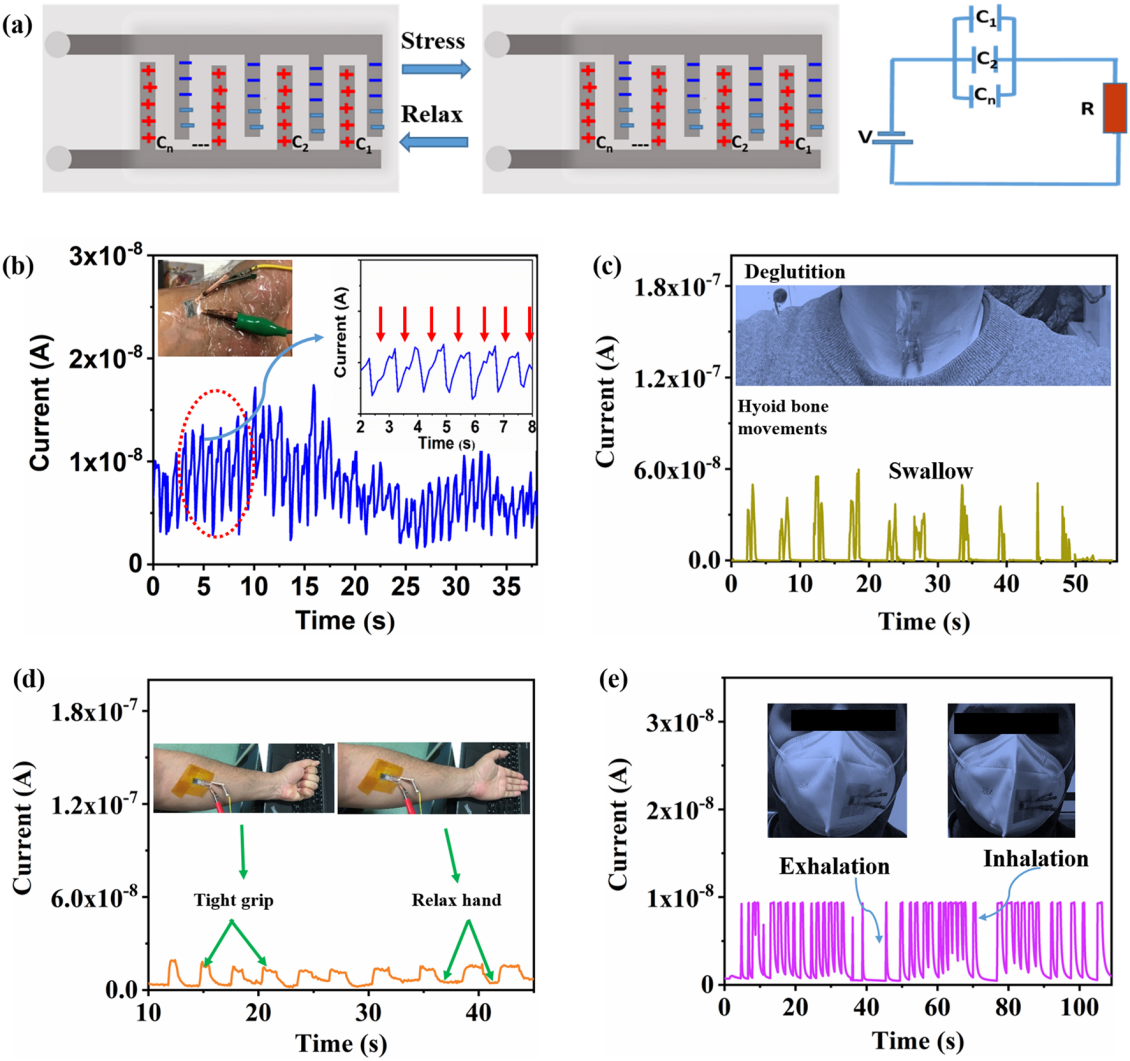
**a** Schematic of working mechanism for flexible PPy-CNT@rGO microsensor, **b** Current variation of micro-strain sensor with wrist pulse, and the inset shows the zoom view of the initial few pulsating electrical waves. **c** Current variation with hyoid bone movement to observe normal swallowing function. **d** Arm muscle (flexor) contraction and relaxation. **e** Pressure variations detection during breathing

Figure 7a proposes the operation mechanism of pressure-sensing for pseudocapacitive iontronic PPy-CNT@rGO// PPy-CNT@rGO microsensor. With the initial input voltage, the positive and negative charges are consistently disseminated at both ends of interdigitated PPy-CNT@rGO//PPy-CNT@rGO electrode, respectively. Owing to the admirable capability of pseudocapacitive combination electric double-layer capacitance of PPy-CNT@rGO electrode, the positive and negative electrolyte ions are evenly distributed at the interface between electrolyte and PPy-CNT@rGO active material. Hence, the neighboring interspace electrode and PVA/PSA gel electrolyte develop a microsupercapacitor unit Ci, the PPy-CNT@rGO// PPy-CNT@rGO microstructure is equivalent to sensor composed of capacitors C1, C2, …, and Cn in parallel. The corresponding diagram of the equivalent circuit for the sensor is shown on the right of Fig. 7a, where the electrode resistance *R* is considered in the circuit, and the applied voltage of the capacitor acts as the power supply *V*. When a definite amount of pressure is applied on the sensor, internal microstructure of PPy-CNT@rGO active materials is distorted due to the external pressure. During the stress, the gel dielectric layer fills the channels between the fingers of the interdigitated PPy-CNT@rGO electrode due to deformation. Moreover, the close contact of porous PPy-CNT increases the electron transmission path and sufficient interface between active material and electrolyte, reducing interface contact resistance and charge transfer resistance. Under the action of an improved interior electric field inside PPy-CNT@rGO intervened electrode, more electrolyte cations and anions separate and collect at the electrode/electrolyte interface. The effect of enhanced pseudocapacitance and electric double layer produces current response toward external pressure.

One of the problems with traditional capacitive sensor is the detection of weak mechanical signals of pressure less than 1 Pa. According to the equation of sensitivity below, the sensitivity of a pressure sensor is related to the area of electrodes and the relative distance between the electrodes.

3 $$S = \left( {\frac{A}{{A_{{\text{o}}} }} \times \frac{{d_{{\text{o}}} }}{d} \times \frac{{ \in_{{\text{r}}} }}{{ \in r_{{\text{o}}} }}} \right) - 1 \times \frac{1}{\Delta P}$$

By changing one of the values of *A*, *d*, or *ϵ* or simultaneously, the sensitivity will be improved. As-prepared flexible pressure sensor possesses an interdigital distance of 100 µm with flexibility to the tensile strain resulting in large variation in signals with the minimum time. To detect the bio signals, the sensor is kept on human wrist and the pulse beats are recorded in the form of variation in current signals at a low operating voltage of 50 mV. The number of pulse oscillations recorded in 35 s is 42, which is with uniform interval (Fig. 7b).

In order to further explore the application of sensors in wearable Eskin sensing applications, hyoid bone movement in the swallowing process was detected by flexible microsensor. To observe normal hyoid bone movement, physicians conduct X-ray video fluoroscopic swallowing tests, which even though is a standard technique, still have limitations such as exposure to radiation and high cost. The flexible microsensor is capable of tracking the hyoid bone movement by attaching the sensor to the surface of the human neck. Figure 7c displays the current time profile of the sensor attached to the user’s neck. During deglutition, the hyoid bone moves up and down sequentially and exerts stress on the microsensor. The change in stress is observed as a variation in electrical signals. Such a sensor-supported approach leads toward a substitute and widely available technique for online hyoid bone motion tracking without any harmful radiation side effects, and offers a distinct and flexible approach for diagnosing dysphagia and other deglutition disorders.

Moreover, to investigate the biosignal sensing capability, the microsensor is attached to the arm flexors. The current signals increase with the muscle expansion caused by making a tight fist grip, and then came back to the original state after relaxing the hand with muscle contraction (Fig. 7c). The results validate the potential application of flexible microsensor in directing the physical training and recovery of muscle injury by detecting the current variations through the movement of body parts. Finally, the pressure sensor was utilized for the detection of breathing. As seen in Fig. 7d, the pressure sensor outputs a stable and regular sensing pattern toward natural breathing. The sensor is pasted on one side of the mask to sense the inhalation and exhalation. During exhalation, the pressure is exerted on the mask membrane and ultimately on the sensor resulting in an increase in the output current. In the inhalation process, the pressure is released and the current lowers to its original position. The results manifest that the MSC-derived strain sensor can detect small mechanical distortions and can be utilized to sense motion of organs with tiny vibrations.

## Conclusions

In summary, an effective strategy is proposed to amplify the electrochemical performance of an innovative PPy-CNT composite co-electrodeposited on rGO@Au microelectrodes for high-performance PPy-CNT@rGO symmetric MSCs. Among the various conducting polymers based MSCs, the PPy-CNT@rGO MSC displays the best electrochemical performances in areal specific capacitance (65.9 mF cm^−2^ at 0.1 mA cm^−2^), specific energy (5.9 µWh cm^−2^), and specific power (0.4 mW cm^−2^) for planar on-chip MSC, while a high areal capacitance of 70.25 mF cm^−2^ for current collector free flexible MSC. Furthermore, the MSC displays outstanding cycling stability with just 21% capacitance loss after 10,000 charge/discharge cycles at a very high current density of 5 mA cm^−2^, much higher than those of PPy-based previously reported MSCs. Moreover, by transferring microelectrodes on a flexible PDMS elastomers, a highly sensitive capacitive micro-strain sensor is fabricated. The MSC-derived flexible pressure/strain sensor displays response and recovery time of 0.9 and 2 ms, respectively, and can detect biosignals and motion of organs. This work validates that the prepared MSC has auspicious potential in on-chip micro-energy storage, and skin-compatible micro-capacitive sensors for biosignal detection.

### Supplementary Information

Below is the link to the electronic supplementary material.

Supplementary file1 (PDF 1671 kb)
