# Supplementary material for: Pushing the Electrochemical Performance Limits of Polypyrrole Toward Stable Microelectronic Devices
Source: Nanomicro Lett. 2023 Feb 13;15:49. doi: 10.1007/s40820-023-01027-3 (PMC9925634; doi:10.1007/s40820-023-01027-3)
Supplement: Supplementary file 1 — Supplementary file1 (PDF 1671 kb) [file 40820_2023_1027_MOESM1_ESM.pdf]

Supporting Information for

Pushing the Electrochemical Performance Limits of Polypyrrole Toward Stable Microelectronic Devices

Muhammad Tahir[1], Liang He[2, 3, *], Lihong Li[1, *], Yawei Cao[1, 4], Xiaoxia Yu[1, 4], Zehua Lu[1, 4], Xiaoqiao Liao[2], Zeyu Ma[2], Yanlin Song[1, *]

[1]Key Laboratory of Green Printing, CAS Research/Education Centre for Excellence in Molecular Sciences, Institute of Chemistry Chinese Academy of Sciences (ICCAS), Beijing 100190, P. R. China

[2]School of Mechanical Engineering, Sichuan University, Chengdu 610065, P. R. China

[3]Med+X Center for Manufacturing, West China Hospital, Sichuan University, Chengdu 610041, P. R. China

[4]School of Chemistry and Biological Engineering University of Science and Technology Beijing, Beijing 100083, P. R. China

*Corresponding authors. E-mail: hel20@scu.edu.cn (Liang He), lilihong1209@iccas.ac.cn (Lihong Li), ylsong@iccas.ac.cn (Yanlin Song)

S1 Mathematical Calculations

ESR values are calculated from the discharge plot at a current density of 0.1 mA cm^{-2} by Eq. (S1).

$$\text{Effective series resistance: (ESR)} = \frac{iRdrop}{2I} \quad \text{(S1)}$$

Where iR is the voltage drop at the starting of the discharge curve and I is the discharge current.

Areal cell capacitance (C_{area}) is calculated from charge-discharge curves according to the equations (S2).

$$\text{Areal capacitance } (C_a) = \frac{I\triangle t}{A\triangle V} \quad \text{(S2)}$$

Where A is the total area of microelectrodes, I is the applied current, Δt is the discharge time and ΔV is the operating voltage window.

Areal energy density (E_a) and areal power density (P_a) are calculated by equations (S3) and (S4).

$$\text{Areal energy density } (E_a) = \frac{Ca(\triangle V)^2}{7200} \quad \text{(S3)}$$

$$\text{Areal power density } (P_a) = Ea \times 3600/\triangle t \quad \text{(S4)}$$

S2 Supplementary Figures and Tables

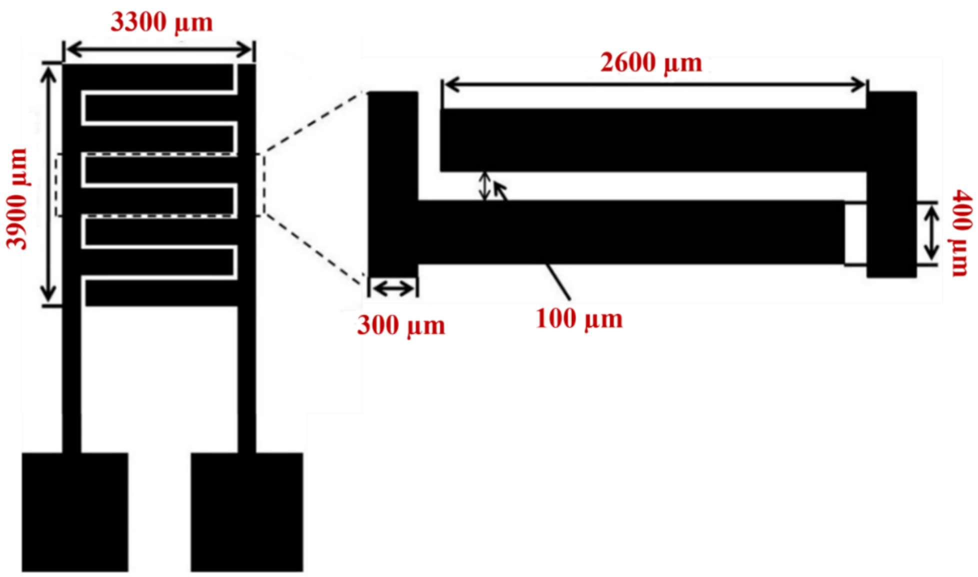

Fig. S1 The design and dimensions of the interdigitated MSCs/microsensors

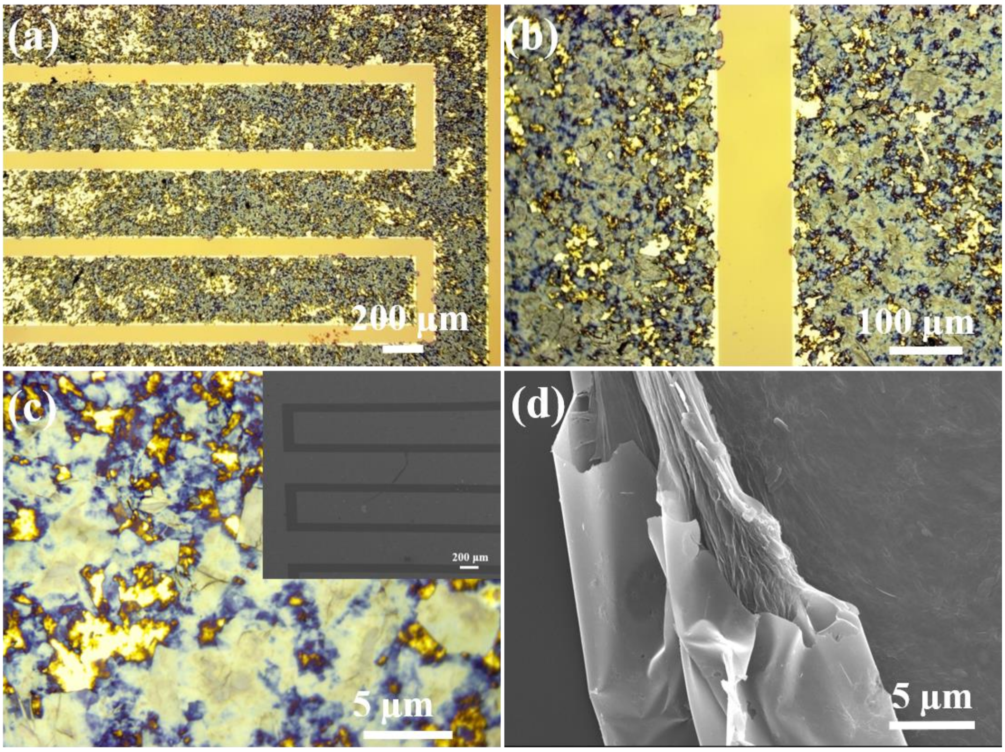

Fig. S2 Electrochemically deposited multilayer rGO on Cr/Au micropatterns. **a, b** Optical microscopic images. **c** High-resolution optical microscopic image, and the SEM image (inset) of rGO deposited on Cr/Au fine micropatterns after lift-off. **d** Cross-sectional SEM image of a channel with Au and rGO

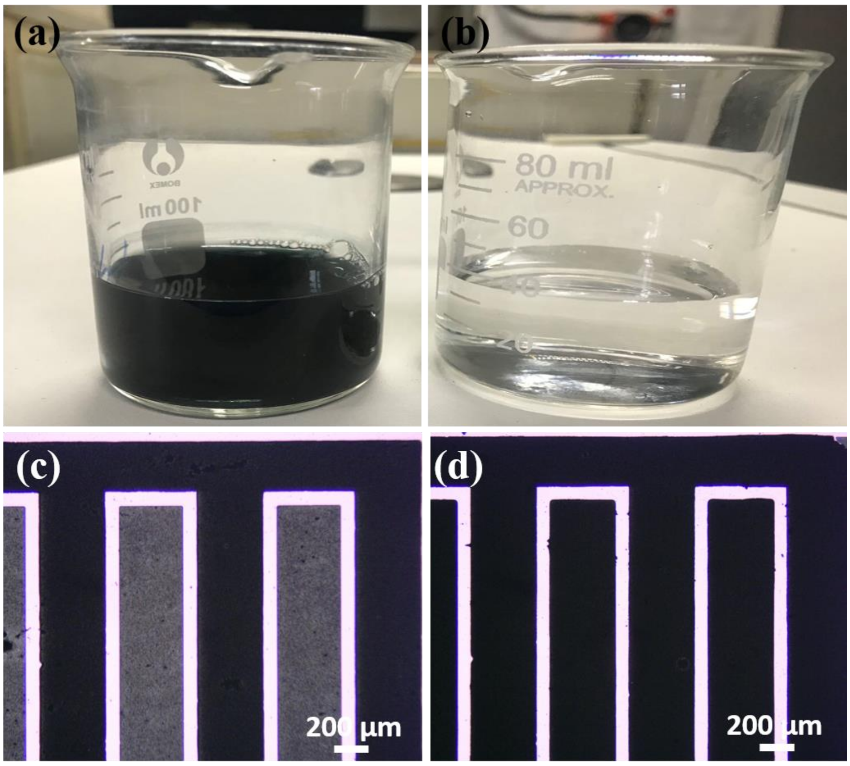

Fig. S3 Optical images of the solutions prepared for electrochemical polymerization. **a** PPy and CNT. **b** PPy (20 minutes after synthesis). **c** Optical microscopic image of rGO@Au micropatterns with one side coated with PPy (deposition time: 60 s) and the other side with PPy-CNT (deposition time: 180 s). **d** Optical microscopic image of PPy-CNT micropatterns (both sides are coated with PPy-CNT)

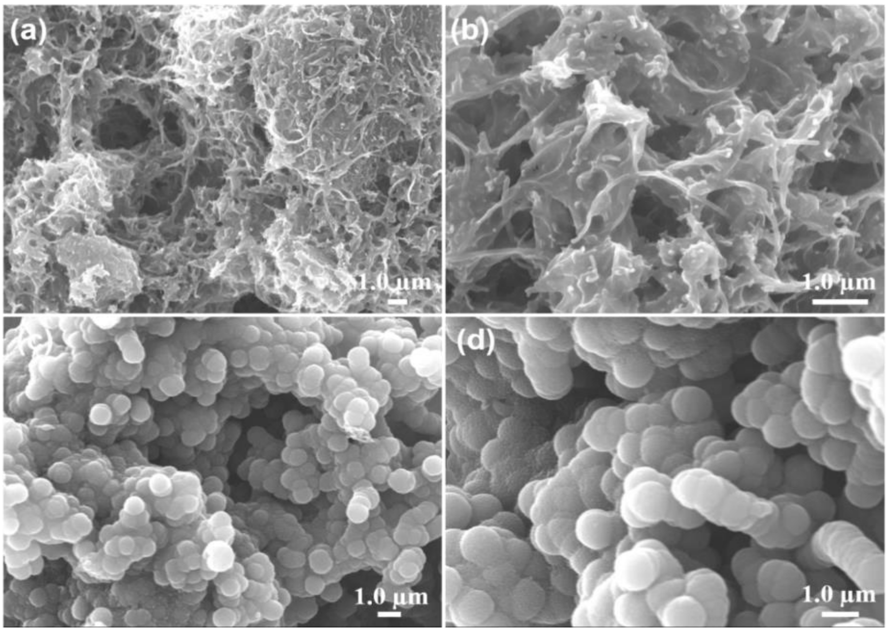

Fig. S4 Low- and high- magnification SEM images of **a, b** PPy-CNT@rGO microelectrodes by deposition of 20 s, and **c, d** deposition of 180 s

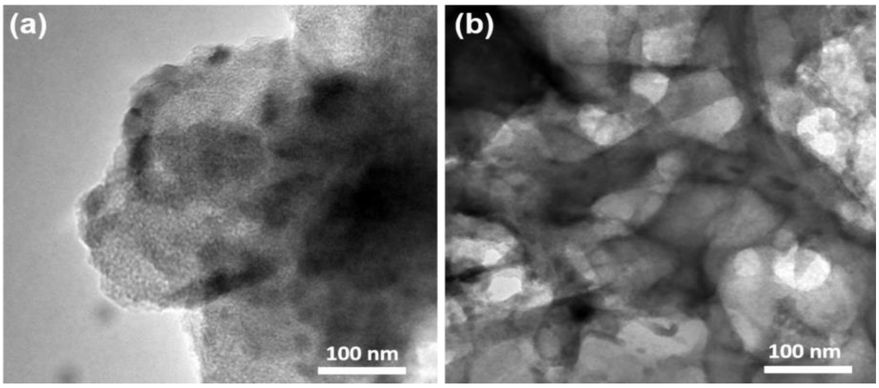

Fig. S5 TEM images of **a** electrodeposited PPy and **b** PPy-CNTs. The embedded CNTs are evenly distributed in the polymer matrix

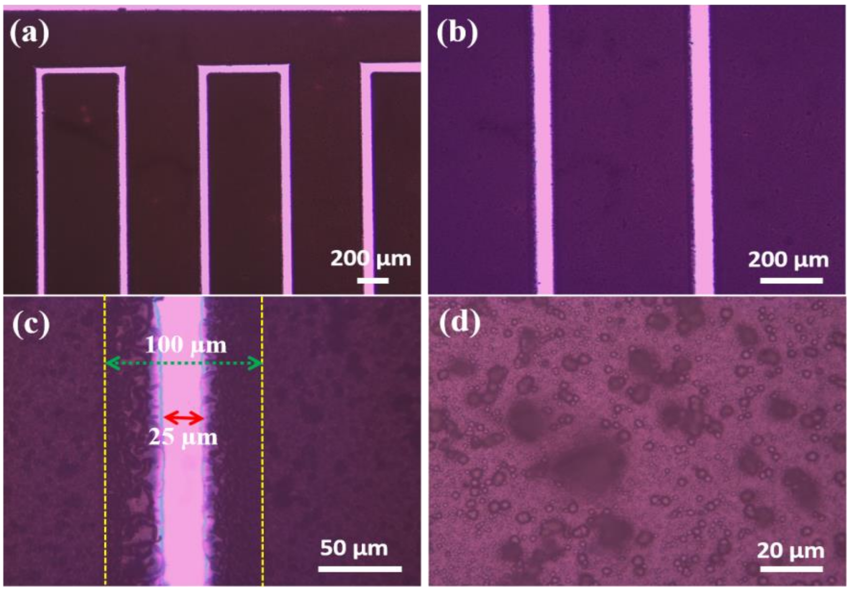

Fig. S6 a-c Optical microscopic images of the PPy deposited on Cr/Au micro current collectors. One-fourth of the interspace is covered with PPy by polymerization (time: 3 minutes). **d** Top-view optical microscopic image of the deposited dense PPy film

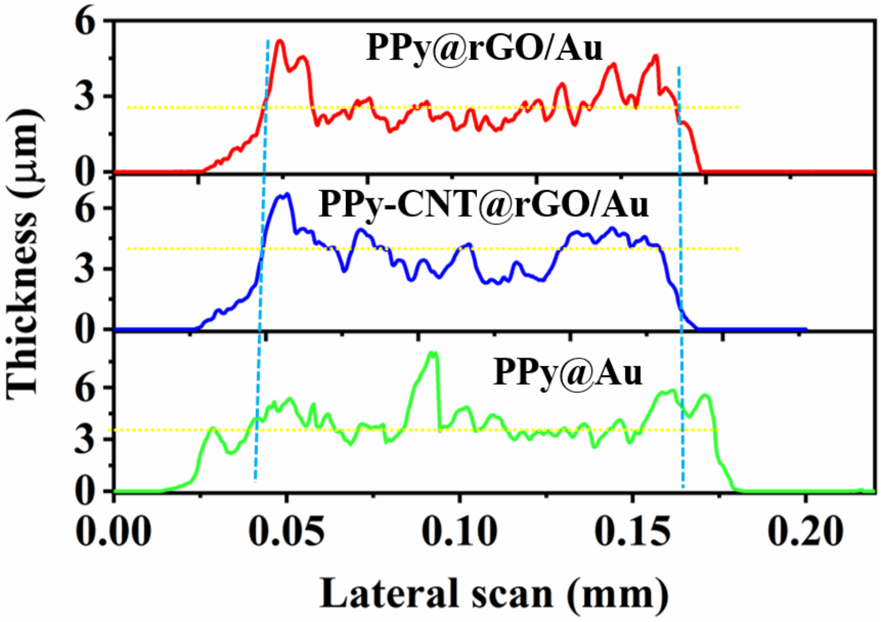

Fig. S7 Height profiles of the microelectrodes obtained from a step surface profiler

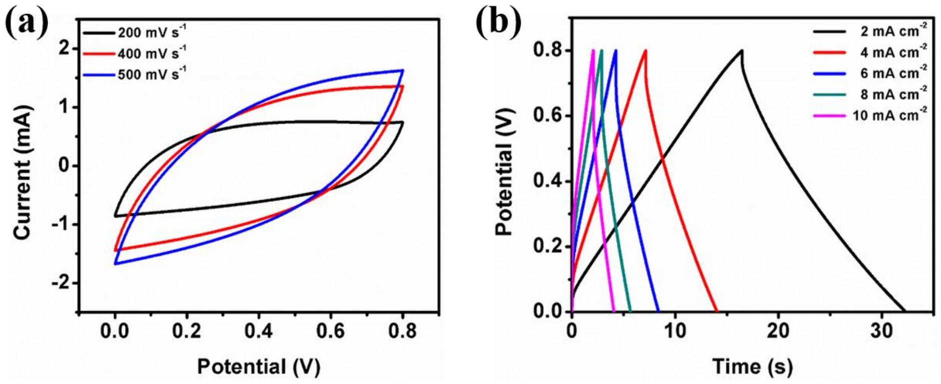

Fig. S8 a CV curves of PPy-CNT@rGO MSC at high scan rates (200-500 mV s^{-1}). **b** GCD curves of PPy-CNT@rGO MSC at high current densities (2-10 mA cm^{-2})

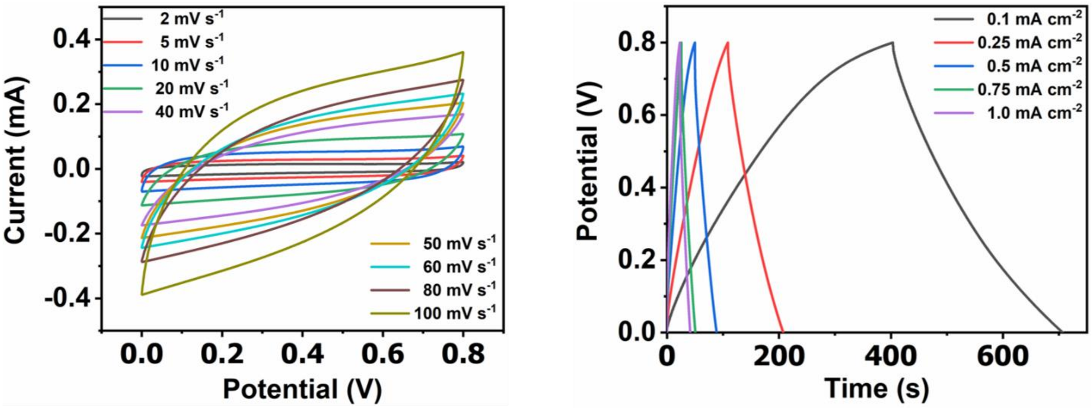

Fig. S9 a CV curves of PPy@Au MSC at different scan rates, and **b** GCD curves of PPy@Au MSC at different current densities

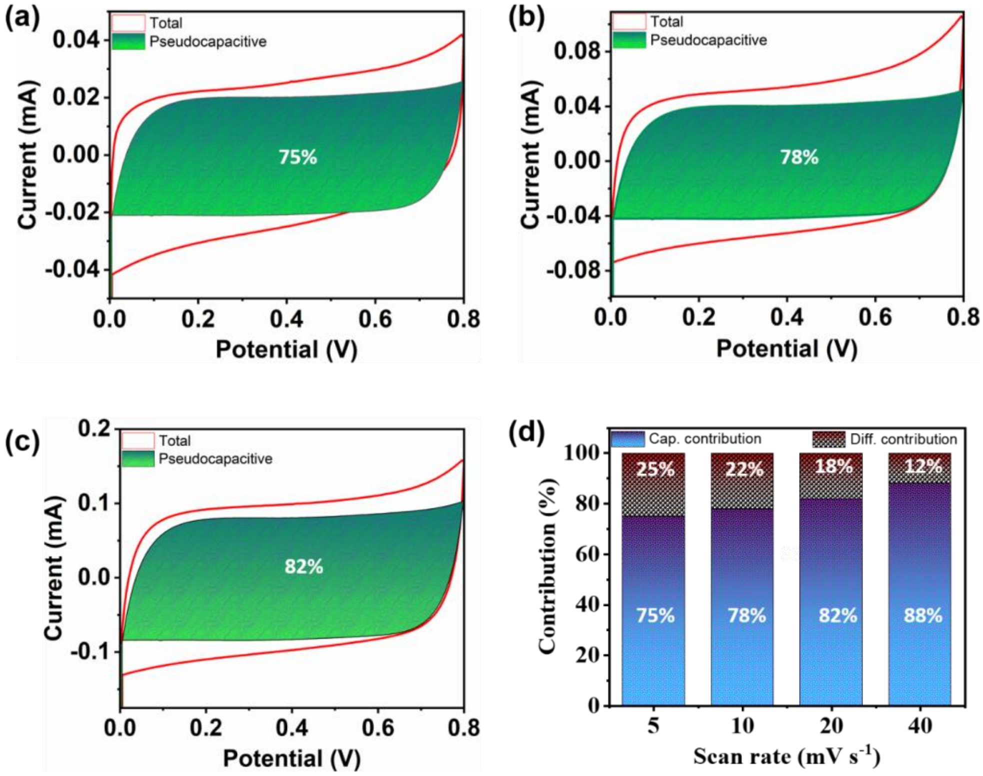

Fig. S10 Capacitive and diffusion-controlled contribution of PPy-CNT@rGO MSC at scan rates of **a** 5 mV s^{-1}, **b** 10 mV s^{-1}, **c** 20 mV s^{-1} and **d** The normalized capacitance and diffusion controlled contribution at distinct scan rates (5, 10, 20, and 40 mV s^{-1})

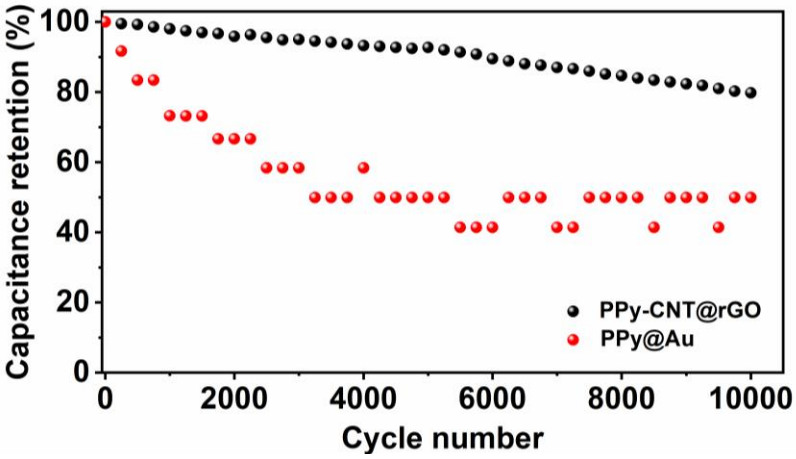

Fig. S11 The cycling performance of PPy-CNT@rGO and PPy@Au

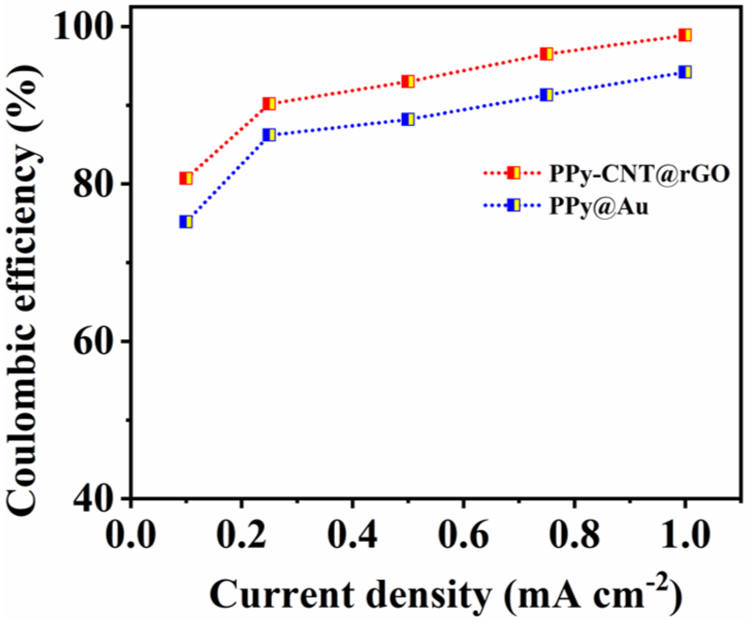

Fig. S12 Coulombic efficiency versus current density of the PPy-CNT@rGO and PPy@Au MSCs

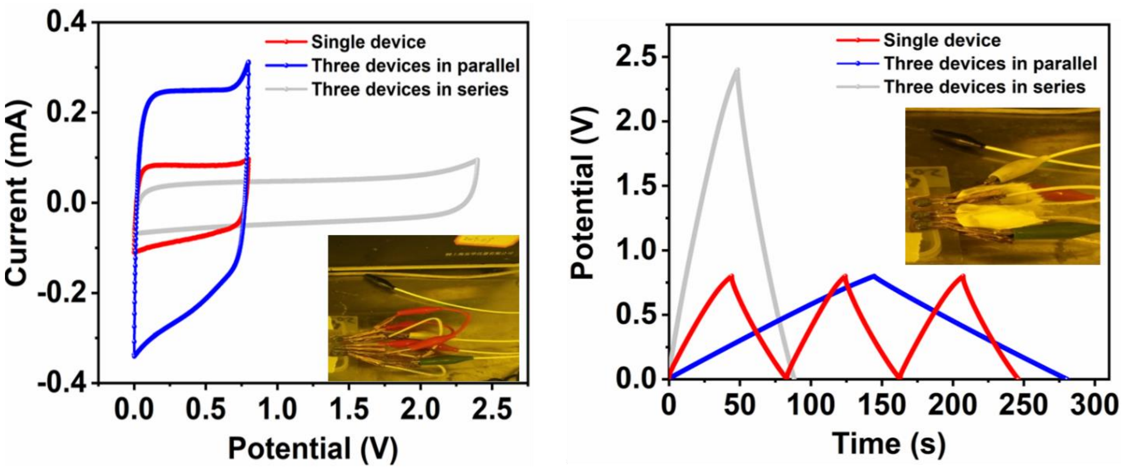

Fig. S13 a CV curves at a scan rate of 50 mV s^{-1} and **b** GCD curves at a current density of 1 mA cm^{-2} of single MSC, series, and parallel connected three MSCs

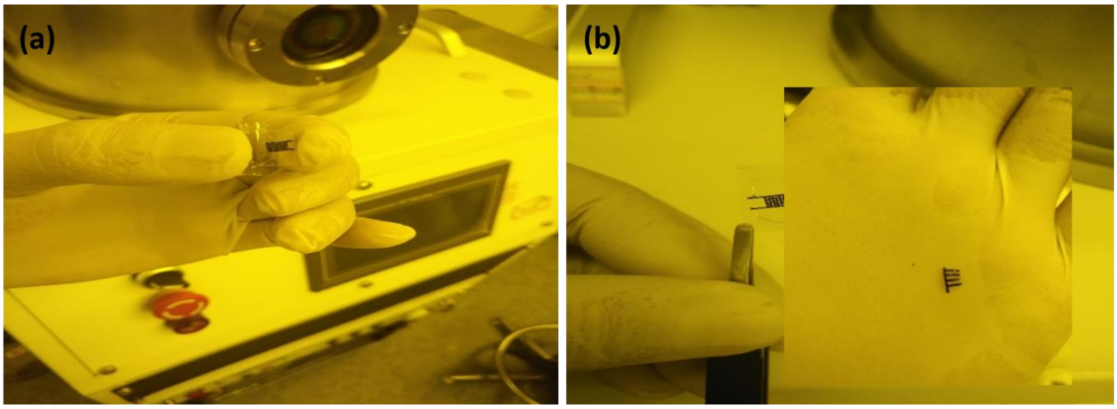

Fig. S14 a, b Optical images of transferred microelectrodes and peeled channels (inset)

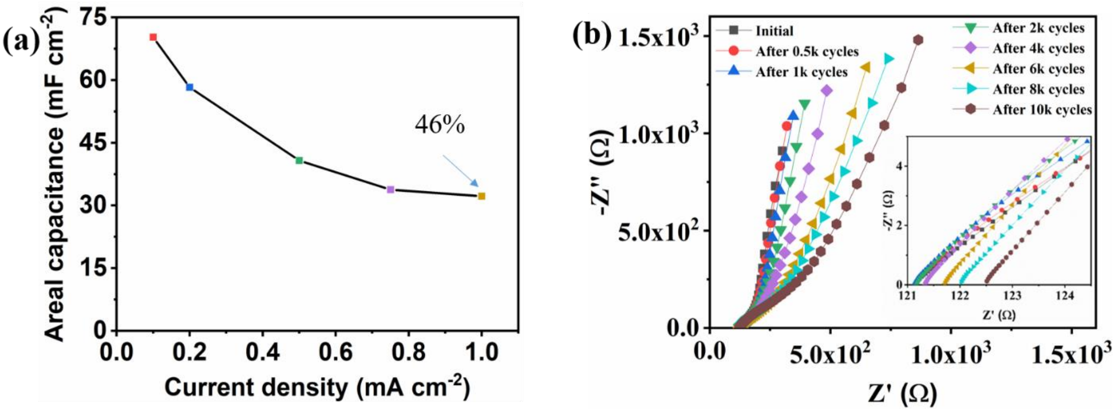

Fig. S15 a Rate performance, **b** In-situ electrochemical impedance spectroscopy (in-situ EIS) of current collector free flexible PPy-CNT@rGO MSC at different cycles

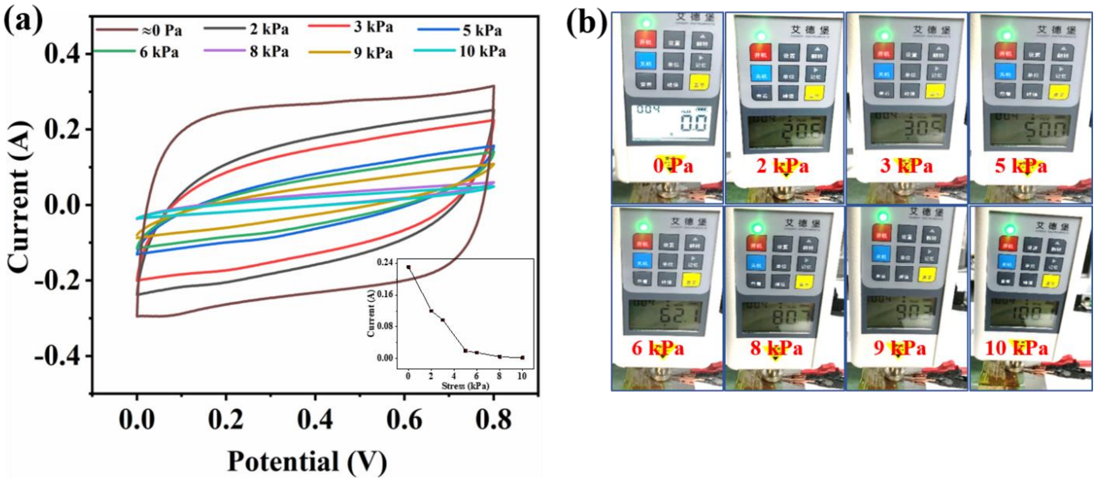

Fig. S16 a CV curves of the PPy-CNT@rGO microelectrodes tested in two electrodes at a scan rate of 40 mV s^{-1} under different applied stress. **b** Digital photographs of the pressure gauge displaying different amount of stress applied on MSC

Table S1 Comparison of electrochemical characteristics of recently reported CPs electrodes and our electrode

**Sample**	**Electrolyte**	***C_A* (mF cm^{-2})**	**Voltage window (V)**	**Energy density (μWh cm^{-2})**	**Cycling performance**
PEDOT@rGO// PPy@rGO-AMSC [S1]	2 M KCl	15.9	0-1.5	5.2	79%/5000 cycles
Graphene/PEDOT [S2]	PVA/H_2SO_4	19.3	0-0.8	2.24	88.6%/5000 cycles
MnO_2/GO/PEDOT [S3]	PVA/H_2SO_4	23.04	0-1.2	7.3	93.1%/5000 cycles
PPy-Si nanowire [S4]	H_2SO_4/PVA	14	1.5	1.3	70%/10000 cycle
PANI-rGO/PDMS [S5]	H_2SO_4/PVA	4.06	0-0.8	1.8 (vol.)	68%/10,000 cycles
Ag@PPy [S6]	H_3PO_4/PVA	47.5	0-0.8	4.33	77.6%/10,000 cycle
MnO_2@Ppy [S7]	PVA/LiCl	13	0-0.8	1.0	84%/5000 cycles
Graphene/PEDOT [S8]	PVA/H3PO4	15.3	0-1.2	1.5	81%/2500 cycles
PEDOT MSC [S9]	PVA/H_2SO_4	9	0-0.8	7.7 (vol.)	80%/1000 cycles
MXene/PEOT [S10]	LiCl/PVA	2.4	0-0.6	1.1	82%/10,000 cycles
Polymer-MXene [S11]	LiCl/PVA	69.5	0-1.6	250 (volumetric)	92%/10,000 cycles
PPy-CNT@rGO*	PVA/H_3PO_4	65.9	0-0.8	5.8	79%/10,000 cycles

Table S2 Comparison of electromechanochemical characteristics of recently reported capacitive sensors and our integrated microsensor

**Sensor Materials**	**Configuration**	**Response Time (ms)**	**Cycles**	**Refs.**
LMs-TPE	Tubular	50	3500	[S12]
Vertical graphene(VGr)	Stacked	180	1000	[S13]
MXene/TiS_2	Interdigitated	1000 to 5000	2500	[S14]
MWCNTs/PVC	Stacked	110	2500	[S15]
MXene/CF	Interdigitated	50	1000	[S16]
PI/CNT aerogel	Stacked	50	1000	[S17]
$Ti_3C_2T_x$-MXene	Stacked	98	10,000	[S18]
PEDOT-CNT@rGO	Interdigitated	0.9	2500	This work

Supplementary References

[S1] M. Tahir, L. He, W. Yang, X. Hong, W.A. Haider et al., Boosting the electrochemical performance and reliability of conducting polymer microelectrode via intermediate graphene for on-chip asymmetric micro-supercapacitor. J. Energy Chem. **49**, 224-232 (2020). https://doi.org/10.1016/j.jechem.2020.02.036

[S2] W. Yan, J. Li, G. Zhang, L. Wang, D. Ho, A synergistic self-assembled 3D PEDOT:PSS/graphene composite sponge for stretchable microsupercapacitors. J. Mater. Chem. A **8**(2), 554-564 (2019). https://doi.org/10.1039/C9TA07383C

[S3] H.U. Lee, C. Park, J.H. Jin, S.W. Kim, A stretchable vertically stacked microsupercapacitor with kirigami-bridged island structure: MnO_2/graphene/Poly (3, 4-ethylenedioxythiophene) nanocomposite electrode through pen lithography. J. Power Sources **453**, 227898 (2020). https://doi.org/10.1016/j.jpowsour.2020.227898

[S4] D. Aradilla, D. Gaboriau, G. Bidan, P. Gentile, M. Boniface et al., An innovative 3-D nanoforest heterostructure made of polypyrrole coated silicon nanotrees for new high performance hybrid micro-supercapacitors. J. Mater. Chem. A **3**(26), 13978-13985 (2015). https://doi.org/10.1039/C5TA03435C

[S5] S. Park, H. Lee, Y.J. Kim, P.S. Lee, Fully laser-patterned stretchable microsupercapacitors integrated with soft electronic circuit components. npj Asia Mater. **10**(10), 959-969 (2018). https://doi.org/10.1038/s41427-018-0080-z

[S6] L. Liu, Q. Lu, S. Yang, J. Guo, Q. Tian et al., All-printed solid-state microsupercapacitors derived from self-template synthesis of Ag@ PPy nanocomposites. Adv. Mater. Technol. **3**(1), 1700206 (2018). https://doi.org/10.1002/admt.201700206

[S7] W.A. Haider, L. He, H.A. Mirza, M. Tahir, A.M. Khan et al., Bilayered microelectrodes based on electrochemically deposited MnO_2/polypyrrole towards fast charge transport kinetics for micro-supercapacitors. RSC Adv. **10**(31), 18245-18251 (2020). https://doi.org/10.1039/D0RA01702G

[S8] H.U. Lee, S.W. Kim, Pen lithography for flexible microsupercapacitors with layer-by-layer assembled graphene flake/PEDOT nanocomposite electrodes. J. Mater. Chem. A **5** (26), 13581-13590 (2017). https://doi.org/10.1039/C7TA02936E

[S9] N. Kurra, M.K. Hota, H.N. Alshareef, Conducting polymer micro-supercapacitors for flexible energy storage and Ac line-filtering. Nano Energy **13**, 500-508 (2015). https://doi.org/10.1016/j.nanoen.2015.03.018

[S10] J. Li, A. Levitt, N. Kurra, K. Juan, N. Noriega et al., MXene-conducting polymer electrochromic microsupercapacitors. Energy Storage Mater. **20**, 455-461 (2019). https://doi.org/10.1016/j.ensm.2019.04.028

[S11] L. Qin, Q. Tao, A.E. Ghazaly, J. Fernandez-Rodriguez, P.O. Persson et al., High-performance ultrathin flexible solid-state supercapacitors based on solution processable $Mo_{1.33}C$ MXene and PEDOT:PSS. Adv. Funct. Mater. **28**(2), 1703808 (2018). https://doi.org/10.1002/adfm.201703808

[S12] P.J. Cao, Y. Liu, W. Asghar, C. Hu, F. Li et al., A stretchable capacitive strain sensor having adjustable elastic modulus capability for wide-range force detection. Adv. Eng. Mater. **22**(3), 1901239 (2020). https://doi.org/10.1002/adem.201901239

[S13] C. Deng, L. Lan, P. He, C. Ding, B. Chen et al., High-performance capacitive strain sensors with highly stretchable vertical graphene electrodes. J. Mater. Chem. C **8**(16), 5541-5546 (2020). https://doi.org/10.1039/D0TC00491J

[S14] L. Wang, Y. Tang, Y. Li, C. Liu, N. Wei et al., Multifunctional integrated interdigital microsupercapacitors and self-powered iontronic tactile pressure sensor for wearable electronics. ACS Appl. Mater. Interfaces **14**(41), 47136-47147 (2022). https://doi.org/10.1021/acsami.2c15117

[S15] T. Dong, Y. Gu, T. Liu, M. Pecht, Resistive and capacitive strain sensors based on customized compliant electrode: comparison and their wearable applications. Sens. Actuator A Phys. **326**, 112720 (2021). https://doi.org/10.1016/j.sna.2021.112720

[S16] Y. Zheng, R. Yin, Y. Zhao, H. Liu, D. Zhang et al., Conductive MXene/cotton fabric based pressure sensor with both high sensitivity and wide sensing range for human motion detection and e-skin. Chem. Eng. J. **420**, 127720 (2021). https://doi.org/10.1016/j.cej.2020.127720

[S17] X. Chen, H. Liu, Y. Zheng, Y. Zhai, X. Liu et al., Highly compressible and robust polyimide/carbon nanotube composite aerogel for high-performance wearable pressure sensor. ACS Appl. Mater. Interfaces **11**(45), 42594-42606 (2019). https://doi.org/10.1021/acsami.9b14688

[S18] L. Gao, M. Wang, W. Wang, H. Xu, Y. Wang et al., Highly sensitive pseudocapacitive iontronic pressure sensor with broad sensing range. Nano-Micro Lett. **13**, 140 (2021). https://doi.org/10.1007/s40820-021-00664-w
